# Supplementary material for: Modified effect of active or passive smoking on the association between age and abdominal aortic calcification: a nationally representative cross-sectional study
Source: BMJ Open. 2021 Oct 12;11(10):e047645. doi: 10.1136/bmjopen-2020-047645 (PMC8520594; doi:10.1136/bmjopen-2020-047645)
Supplement: Supplementary data [file bmjopen-2020-047645supp001.pdf]

Supplemental data

Supplemental Table 1. Association between age and AAC scores in overall or subgroups by smoking

Models	Overall		Never smoker		Smoker		p for interaction
**AAC-24**	β coefficient (95%CI)	p value	β coefficient (95%CI)	p value	β coefficient (95%CI)	p value	
Crude	0.57 (0.49-0.66)	<0.001	0.49 (0.42-0.57)	<0.001	0.66 (0.53-0.80)	<0.001	0.018
Model 1	0.57 (0.49-0.66)	<0.001	0.50 (0.42-0.58)	<0.001	0.66 (0.52-0.79)	<0.001	0.018
Model 2	0.43 (0.33-0.52)	<0.001	0.37 (0.29-0.44)	<0.001	0.53 (0.38-0.68)	<0.001	0.005
**AAC-8**							
Crude	0.20 (0.17-0.23)	<0.001	0.18 (0.15-0.21)	<0.001	0.22 (0.18-0.26)	<0.001	0.081
Model 1	0.20 (0.17-0.23)	<0.001	0.19 (0.16-0.22)	<0.001	0.22 (0.18-0.26)	<0.001	0.081
Model 2	0.16 (0.12-0.19)	<0.001	0.14 (0.11-0.18)	<0.001	0.18 (0.14-0.23)	<0.001	0.017

β coefficient (95%CI) for AAC scores with a 5-year increase of age. p for interaction was estimated by the survey-weighted Wald test was used to assess the potential interaction between age and smoking for AAC. Model 1 (n=3,140): adjusted for sex, and race/ethnicity. Model 2 (n=2,992): additionally adjusted for body mass index, smoking status, alcohol consumption, physical activity, diabetes, hypertension, TC, TG, HDL-C, eGFR, and lowing lipid agents.

Lv L, *et al. BMJ Open* 2021; 11:e047645. doi: 10.1136/bmjopen-2020-047645

Supplemental Table 2. Association between active smoking status and AAC scores

	Never smoking	Quit smoking	Current smoking	
**AAC-8**		**β (95%CI)**	**β (95%CI)**	**p for trend**
Crude	Reference	0.31 (0.17-0.45)	0.23 (0.04-0.43)	0.002
Model 1	Reference	0.15 (0.01-0.29)	0.40 (0.22-0.58)	<0.001
Model 2	Reference	0.14 (0.00-0.29)	0.29 (0.12-0.46)	0.003
**AAC-24**		**β (95%CI)**	**β (95%CI)**	
Crude	Reference	0.92 (0.55-1.29)	0.51 (0.08-0.94)	0.001
Model 1	Reference	0.46 (0.12-0.79)	0.96 (0.54-1.38)	<0.001
Model 2	Reference	0.46 (0.10-0.81)	0.70 (0.34-1.05)	0.001
**Severe AAC**		**OR (95%CI)**	**OR (95%CI)**	
Crude	Reference	2.02 (1.51-2.71)	1.59 (0.94-2.68)	0.012
Model 1	Reference	1.66 (1.18-2.35)	4.30 (2.40-7.71)	<0.001
Model 2	Reference	1.69 (1.12-2.56)	3.39 (2.04-5.66)	<0.001
**Subclinical AAC**		**OR (95%CI)**	**OR (95%CI)**	
Crude	Reference	1.85 (1.37-2.49)	1.73 (1.12-2.66)	0.003
Model 1	Reference	1.47 (1.05-2.07)	2.78 (1.82-4.25)	<0.001
Model 2	Reference	1.46 (1.00-2.14)	2.06 (1.34-3.16)	0.004

β coefficient (95%CI) for AAC scores acorss the smoking status with never smoking as reference. Model 1 (n=3,140): adjusted for age, sex, and race/ethnicity. Model 2 (n=2,992): additionally adjusted for body mass index, alcohol consumption, physical activity, diabetes, hypertension, TC, TG, HDL-C, eGFR, and lowing lipid agents.

Lv L, *et al. BMJ Open* 2021; 11:e047645. doi: 10.1136/bmjopen-2020-047645

Supplemental Table 3. Association between smoking cessation and AAC scores in smokers

	**Current smoking** OR (95%CI)	**Smoking Cessation <10y** OR (95%CI)	**Smoking Cessation ≥10y** OR (95%CI)	**p for trend**
**Subclinical AAC**	Reference	0.97 (0.63-1.48)	0.65 (0.44-0.94)	0.023
**Severe AAC**	Reference	0.96 (0.40-2.34)	0.37 (0.20-0.69)	0.003

OR (95%CI) for the risk of severe AAC or subclinical AAC was estimated by weighted logistics regression analysis, adjusted for age, sex, race/ethnicity, body mass index, alcohol consumption, physical activity, diabetes, hypertension, TC, TG, HDL-C, eGFR, and lowing lipid agents.

Lv L, *et al. BMJ Open* 2021; 11:e047645. doi: 10.1136/bmjopen-2020-047645

Supplemental Table 4. Association between secondhand smoking exposure (SHS) and AAC in never smoker

SHS	Subclinical AAC OR (95%CI)	p value	Severe AAC OR (95%CI)	p value
self-report	0.96 (0.20-4.71)	0.958	0.61 (0.29-1.30)	0.183
NNAL	0.61 (0.30-1.26)	0.167	0.73 (0.51-1.03)	0.073
Cotinine	0.84 (0.49-1.44)	0.500	0.96 (0.68-1.35)	0.792

SHS exposure, was defined as self-report, urinary NNAL≥0.001ng/mL, or serum cotinine ≥0.015ng/mL. OR (95%CI) for the risk of severe AAC or subclinical AAC was estimated by weighted logistics regression analysis, adjusted for age, sex, race/ethnicity, body mass index, alcohol consumption, physical activity, diabetes, hypertension, TC, TG, HDL-C, eGFR, and lowing lipid agents.
