# Supplementary material for: Modified effect of active or passive smoking on the association between age and abdominal aortic calcification: a nationally representative cross-sectional study
Source: BMJ Open. 2021 Oct 12;11(10):e047645. doi: 10.1136/bmjopen-2020-047645 (PMC8520594; doi:10.1136/bmjopen-2020-047645)
Supplement: Author's manuscript [file bmjopen-2020-047645.draft_revisions.pdf]

BMJ Open is committed to open peer review. As part of this commitment we make the peer review history of every article we publish publicly available.

When an article is published we post the peer reviewers' comments and the authors' responses online. We also post the versions of the paper that were used during peer review. These are the versions that the peer review comments apply to.

The versions of the paper that follow are the versions that were submitted during the peer review process. They are not the versions of record or the final published versions. They should not be cited or distributed as the published version of this manuscript.

BMJ Open is an open access journal and the full, final, typeset and author-corrected version of record of the manuscript is available on our site with no access controls, subscription charges or pay-per-view fees (http://bmjopen.bmj.com).

If you have any questions on BMJ Open's open peer review process please email info.bmjopen@bmj.com

BMJ Open

Age-related abdominal aortic calcification is modified by smoking exposure in adults aged 40-80 years, a nationally representative cross-sectional study

Journal:	*BMJ Open*
Manuscript ID	bmjopen-2020-047645
Article Type:	Original research
Date Submitted by the Author:	08-Dec-2020
Complete List of Authors:	Lv, Luyan; Qujing Normal University, Department of Geriatrics, Qujing First People's Hospital<br>Wu, Shixian; Qujing Normal University<br>Yang, Yungui; Qujing Normal University, Department of Geriatrics<br>yue, xiongli; Qujing Normal University, Department of Geriatrics, Qujing First People's Hospital
Keywords:	Cardiology < INTERNAL MEDICINE, Cardiovascular imaging < RADIOLOGY & IMAGING, Vascular medicine < INTERNAL MEDICINE

SCHOLARONE™
Manuscripts

I, the Submitting Author has the right to grant and does grant on behalf of all authors of the Work (as defined in the below author licence), an exclusive licence and/or a non-exclusive licence for contributions from authors who are: i) UK Crown employees; ii) where BMJ has agreed a CC-BY licence shall apply, and/or iii) in accordance with the terms applicable for US Federal Government officers or employees acting as part of their official duties; on a worldwide, perpetual, irrevocable, royalty-free basis to BMJ Publishing Group Ltd ("BMJ") its licensees and where the relevant Journal is co-owned by BMJ to the co-owners of the Journal, to publish the Work in this journal and any other BMJ products and to exploit all rights, as set out in our licence.

The Submitting Author accepts and understands that any supply made under these terms is made by BMJ to the Submitting Author unless you are acting as an employee on behalf of your employer or a postgraduate student of an affiliated institution which is paying any applicable article publishing charge ("APC") for Open Access articles. Where the Submitting Author wishes to make the Work available on an Open Access basis (and intends to pay the relevant APC), the terms of reuse of such Open Access shall be governed by a Creative Commons licence – details of these licences and which Creative Commons licence will apply to this Work are set out in our licence referred to above.

Other than as permitted in any relevant BMJ Author's Self Archiving Policies, I confirm this Work has not been accepted for publication elsewhere, is not being considered for publication elsewhere and does not duplicate material already published. I confirm all authors consent to publication of this Work and authorise the granting of this licence.

For peer review only

Age-related abdominal aortic calcification is modified by smoking exposure in adults aged 40-80 years, a nationally representative cross-sectional study

Brief title:Vascular calcification, age, and smoking

Luyan Lv[1†], Shixian Wu[2†], Yungui Yang[1*], Xiongli Yue[1*]

[1]Department of Geriatrics, Qujing First People's Hospital, Qujing, China;

[2]Ministry of Science and education, Qujing First People's Hospital, Qujing, China;

[†] contributed equally to this article.

Address for correspondence: Xiongli Yue, Yuanlin Road, Qilin District, Qujing, China, 655000, Tel: +86 087 4331 9938, Email: yxl13529891626@163.com;Yungui Yang, Yuanlin Road, Qilin District, Qujing, China, Tel: +86 087 4331 9938, Email: 1369687326@qq.com.

Table number: 2

Figure number: 3

For peer review only - http://bmjopen.bmj.com/site/about/guidelines.xhtml

Abstract:

Objective: Arterial calcification was a critical mark of the atherosclerotic process in aging cardiovascular disease. Given the deleterious effects of smoking on atherosclerosis, this study tested the hypothesis that the association of age with vascular calcification was modified by smoking exposure.

Methods: A cross-sectional analysis was conducted in 3140 individuals aged 40-80 years in NHANES 2013-2014. Abdominal aortic calcification score was determined by dual-energy X-ray absorptiometry (DXA) scans. Multiple linear regression models were used to assess the association between age and aortic calcification. Survey-weighted Wald test was used to evaluate the potential interaction.

Results: Abdominal aortic calcification (AAC) was positively associated with age in the general population. After adjustment for age, sex, race/ethnicity, and other cardiovascular risk factors, the β coefficient for scores of AAC-24 was 0.43 (95CI% 0.33-0.52, p<0.001) per each 5-year increase in age. As expected, the β coefficients for age-related vascular calcification was especially stronger in smokers than non-smokers (p for interaction =0.005). According to spline fitting, the progression of vascular calcification was significantly put forward to 45 years in smokers compared to that after 60 years in non-smokers. Quit smoking may compromise the deleteriousness for vascellum especially in younger adults. However, the difference for age-related calcification in non-smoker with or without second-hand smoke exposure was minor, regardless of definition by self-report, serum cotinine, or urinary 4-(Methylnitrosamino)-1-(3-pyridyl)-1-butanol.

For peer review only - http://bmjopen.bmj.com/site/about/guidelines.xhtml

Conclusions: Smoking significantly accelerates the progression of age-related subclinical atherosclerosis. Early quit smoking should be strengthened among young smokers. The effect of passive smoking exposure on arteriosclerosis should be strictly assessed.

Ethics and dissemination

This study was conducted in accordance with the Declaration of Helsinki. The protocols of NHANES were approved by the National Center for Health Statistics ethics review board. Informed consent was obtained from all participants.

Keywords:

Age, aortic calcification, arteriosclerosis, smoking, second-hand smoking

For peer review only

Strengths and limitations of this study

1. This study was a nationally representative sample of the adult population of US, suggesting a well generalization of the findings.
2. Data was collected with standardized and validated protocols, suggesting a favorable repeatability.
3. This study evaluated the potential modification for age-related abdominal aortic calcification by active smoking or second-hand smoking exposures, defined by self-reports or nicotine metabolites.
4. This study was based on a cross-sectional design that was less likely to conclude the causality.
5. AAC measurement was only carried out in a population aged 40 years or older. Whether smoking influences age-related vascular calcification in adults less than 40 years old that warrants investigation.

For peer review only

For peer review only - http://bmjopen.bmj.com/site/about/guidelines.xhtml

For peer review only

For peer review only - http://bmjopen.bmj.com/site/about/guidelines.xhtml

Introduction

Cardiovascular disease (CVD) has been the leading cause of death since the early 20th century worldwide, bringing a heavy burden to public health and care[1]. Atherosclerosis has always been the primary pathological basis of CVD among the wide array of underlying cardiovascular lesions[2]. To early identify populations at high risk of subclinical atherosclerosis is essential to prevent the progression of CVD[3]. Abdominal aortic calcification (AAC) was considered as a key hallmark of atherosclerosis and an independent predictor of subsequent cardiovascular morbidity and mortality[1,4]. Accumulated evidence suggested that severe AAC is associated with a higher risk of major cardiovascular events, reflecting poor cardiovascular health status[5].

Epidemiological studies before decades had demonstrated that vascular calcification or atherosclerosis was a common aging-related process [6,7]. Along with the improvement of the primary and second prevention strategies for cardiovascular events, how the association between age and aortic calcification during the contemporary era is unknown. Cardiovascular risk factors might alter the pattern of association of aging with vascular calcification. Smoking is a well-established risk factor for atherosclerosis and CVD via several underlying pathways[8], and has been a critical environmental hazard for public health[9]. It is noteworthy that, approximately 11% of cardiovascular death is attributed to smoking exposure worldwide[10]. Furthermore, secondhand smoke (SHS) exposure in nonsmokers was also associated with cardiovascular disease and other health consequences[11].

For peer review only - http://bmjopen.bmj.com/site/about/guidelines.xhtml

However, the potential interaction between age and active or passive smoking on vascular calcification was unclear. To identify the correlation pattern may provide new insights into the process of atherosclerosis and its early prevention. In this context, this study tested the hypothesis that the association between age and abdominal aortic calcification differs according to smoking exposure in a nationally representative sample of the middle-aged and elderly population.

For peer review only

For peer review only - http://bmjopen.bmj.com/site/about/guidelines.xhtml

Methods

Study Population

The data were extracted from National Health and Nutrition Examination Surveys (NHANES) 2013-2014, an ongoing nationally representative, stratified, multistage probability-sampling survey designed by the Center for Disease Control and Prevention of the United States. The methods and protocols had been described in previous literatures[12,13]. In NHANES 2013-2014, dual-energy X-ray absorptiometry (DXA) scans were conducted in participants aged 40 years and older. Pregnant females were excluded. All those with eligible AAC scores were included in this study (n = 3140). The protocols of NHANES were approved by the National Center for Health Statistics ethics review board. Informed consent was obtained from all participants. Detailed information is available on the website http://www.cdc.gov/nchs/nhanes/irba98.htm.

Abdominal Aortic Calcification Measurement

The scores of abdominal aortic calcification were calculated from a lateral scan of the lumbar spine (vertebrae L1-L4) that was acquired on Hologic Discovery model A densitometers (Hologic, Inc., Marlborough, Massachusetts), using software Apex 3.2 version[1,14]. The radiation exposure from dual-energy X-ray absorptiometry (DXA) for the lateral spine scan was extremely low (less than 20 uSv). All scans were viewed using Optasia SpinAnalyzer software. AAC-24 (Kauppila score) semi-quantitative score was applied for the primary analysis, and AAC-8 score (Schousboe score) was used for sensitive analysis. Both methods had been validated with favorable

For peer review only - http://bmjopen.bmj.com/site/about/guidelines.xhtml

sensitivity and specificity to reflect the severity of abdominal aortic calcification. The DXA examinations were administered by trained and certified radiology technologists. The detailed protocols have been presented in previous studies.

Demographic, clinical, and laboratory variables

Data concerning age, sex, race/ethnicity, smoking status, physical activity, and alcohol consumptions, history of the disease was collected during personal interviews using standardized questionnaires[12]. Physical examination was conducted in the Mobile Examination Centre (MEC) with standardized protocols. Peripheral blood was commenced were collected in MEC and determined in the central laboratory following the standardized procedures. Race/ethnicity was categorized into 4 groups: non-Hispanic white, non-Hispanic black, Hispanic-Mexican, and others. Physical activity was categorized as vigorous, moderate, and less activity as presented elsewhere. Drinking was assessed via questionnaires for the preceding year. One drink was defined as 12-oz beer, 4-oz wine, or 1 ounce of liquor, each covering about 10 g of alcohols[15]. Body mass index (BMI) was presented as weight divided by height squared (kg/m2). Triglyceride (TG), total cholesterol (TC), high-density lipoprotein-cholesterol (HDL-C), and serum creatinine were determined in subjects with morning fasting status. The estimated glomerular filtration rate (eGFR) was calculated by the Chronic Kidney Disease Epidemiology Collaboration (CKD-EPI). Hypertension was defined as antihypertensive administration, systolic BP ≥140

For peer review only

mmHg, or diastolic BP ≥90 mmHg. Diabetes was defined as anti-hyperglycemic therapy or HbA1c ≥6.5%[15].

Smoking was defined as smoke at least 100 cigarettes during his or her life, ascertained through self-reports including both past and current cigarette smoking status. Tobacco metabolites, serum cotinine, and urinary 4-(Methylnitrosamino)-1-(3-pyridyl)-1-butanol (NNAL) were considered as biomarkers of passive smoking exposures in nonsmokers[16]. Serum cotinine and urinary NNAL were determined by high-performance liquid chromatography-tandem mass spectrometric (HPLC-MS) method, with atmospheric pressure chemical ionization or electrospray ionization, respectively. Biosample was pretreated with methyl-D3-cotinine or 4-(Methylnitrosoamino)-1-(3-pyridyl)-1-butanol-1,2',3',4',5',6'-13C^6 as the internal standard. C18 HPLC column was used to separate the samples. The eluent from these injections was monitored by a mass spectrometer. The m/z 80 product ion from the m/z 177 quasi-molecular ion was used to quantify serum cotinine.

Statistical Analysis

Continuous and categorical variables are expressed as mean (SE) and percentage, weighted with primary sampling unit, pseudo-strata, and appropriate sampling weights to account for the complex sampling design. Crude and multivariable linear regression models were applied to assess the association of age with scores of abdominal aortic calcification. Two adjusted models were adopted. Model 1 was adjusted for age, sex, and race/ethnicity. Model 2 was additionally adjusted for body mass index, smoking status, alcohol consumption, physical activity, diabetes,

For peer review only - http://bmjopen.bmj.com/site/about/guidelines.xhtml

hypertension, total cholesterol, HDL-C, estimated glomerular filtration rate, and lowing lipid agents, ACEI/ARBs, and β-blocker administration. The survey-weighted Wald test was used to assess the potential interaction between age and smoking for AAC. The β coefficient (95%CI) was presented as the increased scores of AAC-24 or AAC-8 for each 5-year increase. Two-way fractional polynomial regression spline plots were used to visualize the relationship between age and vascular calcification among smokers or nonsmokers, and SHS or non-SHS. Statistical analysis was conducted with Stata (version SE15.0). All tests with a 2-sided $P<0.05$ were considered statistically significant.

For peer review only

For peer review only - http://bmjopen.bmj.com/site/about/guidelines.xhtml

Results

Overall, 3140 adults aged ≥40 years had measured abdominal aortic calcification in NHANES 2013-2014 (**Table 1**). The weighted mean age was 57.4 years and 51.9% (n= 1622) were female. There are 54.0% non-smokers and 46.0% smokers, including 28.2% ever smoking and 17.8% current smoking, according to self-report. Compared to those age ≤ 65 years, elder participants had higher proportions of non-Hispanic whites, quit smoking, diabetes, and cardiovascular medications, as well as less physical activity and lower renal function.

The association between age and abdominal aortic calcification in all participants or stratified by smoking was shown in **Table 2**. In univariable and sex- and race-adjusted linear regression models, age was significantly associated with the severity of AAC, with a 0.57 increased scores of AAC-24 for each 5-year increase in age (β coefficient 0.57, 95CI% 0.49-0.66, p<0.001). After adjustment for sex, race, smoking, physical activity, alcohol intakes, BMI, hypertension, diabetes, TC, TG, HDL-C, eGFR, lowering-lipid agents, ACEI/ARB, and β-blockers medications, the association of age and AAC remained significant (β coefficient: 0.43 95CI% 0.33-0.52 for each 5-year increase in age, p<0.001).

Because smoking cigarettes was a strong risk factor for vascular injury, we assessed age-related aortic calcification in subgroups by smoking status. As expected, the effect modification by smoking was observed (p for interaction = 0.005). In fully adjusted models, the β coefficients (95%CIs) per each 5-year increase of age for AAC-24 were 0.37 (0.29-0.44) among participants with non-smoking versus 0.53

For peer review only - http://bmjopen.bmj.com/site/about/guidelines.xhtml

(0.38-0.68) among participants with smoking. That suggested that ever and current smoking may increase age-related abdominal aortic calcification by 30% compared to that in non-smokers. The repeated analysis was performed using AAC-8 scores and the correlations were unchanged. According to the fitting splines (**Figure 1**), non-smokers had a flat trend of age-related calcification before 65 years, whereas the calcification was significantly advanced after 45 years in subjects with smoking. Furthermore, compared to current smokers, participants with quit smoking had lower scores of age-related calcification, especially for those aged 50-70 years (**Figure 2**). However, among nonsmokers, secondhand smoking exposure did not significantly affect age-related abdominal aortic calcification (**Figure 3**).

For peer review only - http://bmjopen.bmj.com/site/about/guidelines.xhtml

Discussion

In this nationally representative sample from the US general population, the association between abdominal aortic calcification and age differed depending on smoking status after adjustment for various confounders. Age-related calcification was flat before 60 years, while that was driven by smoking since 45 years. Quit smoking may relatively mitigate the process of age-induced calcification compared to persistent smokers, especially for the population aged 45-70 years. However, the deleterious effect of secondhand smoking on vascular calcification in adults was not observed. Our study is of particular interest because our findings highlight the benefits of non-smoking and early quit smoking education on the process of age-related vascular calcification or atherosclerosis in the contemporary population.

Vascular calcification was considered to be a degenerative and end-stage consequence of aging processes[6]. Arterial calcification was detected in approximately one-fourth in populations at 50 years old, and the proportion subsequently increased to beyond 60% in population aged more than 75 years[7]. Consistently, our results also noted that the scores of AAC were slowly increased in middle-aged persons, and robustly increased in elderly aged ≥ 65 years.

Vascular calcification has been linked with vascular injury and subclinical atherosclerotic disease[14,17]. Vascular wall calcification was a common byproduct of atherosclerotic plaques and thus was considered as a potential marker of subclinical atherosclerosis as well as a simple tool for cardiovascular risk stratification[14]. Although previous reports have highlighted the implications of AAC as a biomarker

For peer review only - http://bmjopen.bmj.com/site/about/guidelines.xhtml

of cardiovascular morbidity and mortality in general adults[18-20], AAC as a modifiable marker in preventing atherosclerosis and cardiovascular risk has received less attention[21]. Our findings suggested quit smoke is a simple and effective means to mitigate the process of arterial calcification and atherosclerosis in the general population, especially in young adults. The change of lifestyle seems to be promising, low-cost, and easy to spread.

It is well-known that smoking is one of the strongest risk factors of coronary calcification and atherosclerosis[22-24], whereas the data on smoking and abdominal arterial calcification were limited[14]. Jung and colleagues observed the positive association between AAC and long-term smoking exposure in 218 men aged 40 to 81 years. Even a low dosage of smoking also increased the risk of AAC[25]. Furthermore, Hirooka and colleagues analyzed 313 Japanese and 302 Korean men aged 40-49 years and concluded that smoking with higher pack-years was significantly associated with artery calcium, suggesting a dose-response pattern between cigarette use and progression of atherosclerosis[22]. A small sample size and selection bias may limit the extrapolation.

Based on NHANES data, one recent study noted that serum cotinine levels, a biomarker of smoking, were associated with the risk of severe abdominal aortic calcification in 2840 adults[14]. However, circulating cotinine only reflected short-term exposure of smoking, and the interaction between smoking and age on the process of vascular calcification was unknown. Furthermore, our results highlighted that the process of age-related calcification was slow before 60 years old. By contrast,

For peer review only - http://bmjopen.bmj.com/site/about/guidelines.xhtml

smoking, especially persistent smoking significantly accelerated this process from 40-years old. Early quit smoking may at least part moderate the speed for adults aged ≤70 years. This is the first study that specifically investigates the interaction between age and smoking on the severity of aortic calcification in humans. Notably, previous studies have been established the association between smoking and vascular calcification, suggesting the potential benefits of quit smoking to prevent atherosclerosis[8,14,23,24]. However, the complex interaction with the aging process may be neglected that may provide new insights into the optimal intervention time of atherosclerosis[24]. Our results suggested quit smoking may have little advantage in elderly aged >70 years that highlights the importance of early quitting to reduce the burden of vascular calcification.

Secondhand smoke (SHS) exposure has been proved to be associated with cardiovascular disease and adverse outcomes[11]. About 85% of SHS was derived from side-stream smoke that seems potentially more harmful due to non-filtering[26]. As another purpose, we investigated the relationship between AAC and SHS with the definition by self-report, serum cotinine or urine 4-(methylnitrosamino)-1-(3-pyridyl)-1-butanonol (NNAL) in nonsmokers. However, the neutral link between arterial calcification and passive smoking we noted was not the ultimate conclusion. The questionnaires of secondhand smoking were only designed for the preceding 7 days, although we used the definition of potentially persistent SHS exposure indoors at job or at home. Serum cotinine and urinary NNAL, as more accurate biomarkers of smoking exposure than self-reported, had

For peer review only - http://bmjopen.bmj.com/site/about/guidelines.xhtml

been validated to estimate the SHS exposure of the past 2–4 days and 6–12 weeks, respectively[27]. The relationship was also insignificant in stratifications by cotinine or NNAL. The serum cotinine or urine NNAL only reflected exposures over the past several days or weeks that was insufficient to reflect the long-term exposure. The progression of vascular calcification was a chronic process even beyond the years. Thus, further study may need multiple repeated measurements or questionnaires over years to assess the effect of SHS on age-related calcification.

Limitations and strengths

Our study has some strengths that support a favorable repeatability, including nationally representative of the adult population, standardized and validated protocols, full adjustment for a large number of potential confounders. However, our conclusions should be explained in consideration of the following limitations. First, this study was based on a cross-sectional design that was less likely to conclude the causality. More convincing evidence could be obtained from a longitudinal study with multiple measurements. Second, the analysis for the relationship between secondhand smoking and AAC was not the ultimate seal. The duration of SHS should be considered in further study. Third, AAC measurement was only carried out in a population aged 40 years or older. Whether smoking influences age-related vascular calcification in adults less than 40 years old that warrants investigation. Forth, residual confounding could not be eliminated, though we strictly adjusted for 16 covariates in the regression model.

For peer review only - http://bmjopen.bmj.com/site/about/guidelines.xhtml

Conclusions

Our results indicate that smoking significantly modifies the association between age and the severity of abdominal aortic calcification in adults aged 40 years or older. Age-related vascular calcification is accelerated by smoking that is partly reduced by quit smoking. However, whether secondhand smoking affects this process needs further investigation.

For peer review only

For peer review only - http://bmjopen.bmj.com/site/about/guidelines.xhtml

Acknowledgments: The authors would like to thank all participants and staff in NHANES for their great contributions.

Data availability statement: All data are publicly available on the website http://www.cdc.gov/nchs/nhanes/irba98.htm.

Conflict of interest: The authors declare no potential conflict of interest.

Sources of Funding: This manuscript was written independently. The authors received no financial support.

Author contributions:

Yungui Yang conceived the design of the study. Yungui Yang and Xiongli Yue developed the protocols. Luyan Lv and Shixian Wu organized and analyzed all data. All authors writed and reviewed the manuscript. Yungui Yang and Xiongli Yue take the responsibility for the integrity and accuracy of data.

Patient and public involvement

No patients were involved in the development of the research question or design of the study.

References:

1 Li YW, Chen WL: Clinical relevance of serum uric acid and abdominal aortic-calcification in a national survey. Clin Cardiol 2020

2 Lusis AJ: Atherosclerosis. Nature 2000;407:233-241.

3 Kalsch H, Lehmann N, Moebus S, Hoffmann B, Stang A, Jockel KH, Erbel R, Mahabadi AA: Aortic calcification onset and progression: association with the development of coronary atherosclerosis. J Am Heart Assoc 2017;6

4 Wilson PW, Kauppila LI, O'Donnell CJ, Kiel DP, Hannan M, Polak JM, Cupples LA: Abdominal aortic calcific deposits are an important predictor of vascular morbidity and mortality. Circulation 2001;103:1529-1534.

5 Szulc P: Abdominal aortic calcification: a reappraisal of epidemiological and pathophysiological data. Bone 2016;84:25-37.

For peer review only - http://bmjopen.bmj.com/site/about/guidelines.xhtml

6 Zhang B, Miller VM, Miller JD: Influences of sex and estrogen in arterial and valvular calcification. Front Endocrinol (Lausanne) 2019;10:622.

7 Cannata-Andia JB, Rodriguez-Garcia M, Carrillo-Lopez N, Naves-Diaz M, Diaz-Lopez B: Vascular calcifications: pathogenesis, management, and impact on clinical outcomes. J Am Soc Nephrol 2006;17:S267-S273.

8 Wang Z, Wang D, Wang Y: Cigarette smoking and adipose tissue: the emerging role in progression of atherosclerosis. Mediators Inflamm 2017;2017:3102737.

9 Smoking prevalence and attributable disease burden in 195 countries and territories, 1990-2015: a systematic analysis from the global burden of disease study 2015. Lancet 2017;389:1885-1906.

10 Ezzati M, Henley SJ, Thun MJ, Lopez AD: Role of smoking in global and regional cardiovascular mortality. Circulation 2005;112:489-497.

11 Asfar T, Koru-Sengul T, Ruano-Herreria EC, Sierra D, Lee DJ, Arheart KL: Secondhand smoke exposure among high-risk patients in the united states (nhanes 2001-2012): implications for clinical practice. Nicotine Tob Res 2019;21:551-556.

12 Wang S, Liu Y, Liu J, Tian W, Zhang X, Cai H, Fang S, Yu B: Mitochondria-derived methylmalonic acid, a surrogate biomarker of mitochondrial dysfunction and oxidative stress, predicts all-cause and cardiovascular mortality in the general population. Redox Biol 2020;37:101741.

13 Rossato LT, de Branco F, Azeredo CM, Rinaldi A, de Oliveira EP: Association between omega-3 fatty acids intake and muscle strength in older adults: a study from national health and nutrition examination survey (nhanes) 1999-2002. Clin Nutr 2020

14 Wen X, Xia Y, Guo M, Zhao L, Zhou L: Association between serum cotinine and severe abdominal aortic calcification in us adults. Angiology 2020;71:333-339.

15 Wang S, Tian W, Liu Y, Yan G, Fang S, Wang Y, Yu B: Temporal trend of circulating trans-fatty acids and risk of long-term mortality in general population. Clin Nutr 2020

16 Fu Z, Jiang H, Xu Z, Li H, Wu N, Yin P: Objective secondhand smoke exposure in chronic obstructive pulmonary disease patients without active smoking: the u.s. National health and nutrition examination survey (nhanes) 2007-2012. Ann Transl Med 2020;8:445.

17 Budoff MJ, Achenbach S, Blumenthal RS, Carr JJ, Goldin JG, Greenland P, Guerci AD, Lima JA, Rader DJ, Rubin GD, Shaw LJ, Wiegers SE: Assessment of coronary artery disease by cardiac computed tomography: a scientific statement from the american heart association committee on cardiovascular imaging and intervention, council on cardiovascular radiology and intervention, and committee on cardiac imaging, council on clinical cardiology. Circulation 2006;114:1761-1791.

18 Bastos GF, Voute MT, Hoeks SE, Chonchol MB, Boersma EE, Stolker RJ, Verhagen HJ: Calcification of the abdominal aorta as an independent predictor of cardiovascular events: a meta-analysis. Heart 2012;98:988-994.

19 Martino F, Di Loreto P, Giacomini D, Kaushik M, Rodighiero MP, Crepaldi C, Ronco C: Abdominal aortic calcification is an independent predictor of cardiovascular events in peritoneal dialysis patients. Ther Apher Dial 2013;17:448-453.

20 Bolland MJ, Wang TK, van Pelt NC, Horne AM, Mason BH, Ames RW, Grey AB, Ruygrok PN, Gamble GD, Reid IR: Abdominal aortic calcification on vertebral morphometry images predicts incident myocardial infarction. J Bone Miner Res 2010;25:505-512.

21 Golledge J: Abdominal aortic calcification: clinical significance, mechanisms and therapies. Curr Pharm Des 2014;20:5834-5838.

22 Hirooka N, Kadowaki T, Sekikawa A, Ueshima H, Choo J, Miura K, Okamura T, Fujiyoshi A,

For peer review only - http://bmjopen.bmj.com/site/about/guidelines.xhtml

Kadowaki S, Kadota A, Nakamura Y, Maegawa H, Kashiwagi A, Masaki K, Sutton-Tyrrell K, Kuller LH, Curb JD, Shin C: Influence of cigarette smoking on coronary artery and aortic calcium among random samples from populations of middle-aged japanese and korean men. J Epidemiol Community Health 2013;67:119-124.

23 Rasmussen T, Frestad D, Kober L, Pedersen JH, Thomsen LH, Dirksen A, Kofoed KF: Development and progression of coronary artery calcification in long-term smokers: adverse effects of continued smoking. J Am Coll Cardiol 2013;62:255-257.

24 Lehmann N, Mohlenkamp S, Mahabadi AA, Schmermund A, Roggenbuck U, Seibel R, Gronemeyer D, Budde T, Dragano N, Stang A, Mann K, Moebus S, Erbel R, Jockel KH: Effect of smoking and other traditional risk factors on the onset of coronary artery calcification: results of the heinz nixdorf recall study. Atherosclerosis 2014;232:339-345.

25 Jung JG, Wu LT, Kim JS, Kim ED, Yoon SJ: Relationship between smoking and abdominal aorta calcification on computed tomography. Korean J Fam Med 2019;40:248-253.

26 Park YS, Lee CH, Kim YI, Ahn CM, Kim JO, Park JH, Lee SH, Kim JY, Chun EM, Jung TH, Yoo KH: Association between secondhand smoke exposure and hypertension in never smokers: a cross-sectional survey using data from korean national health and nutritional examination survey v, 2010-2012. Bmj Open 2018;8:e21217.

27 Fu Z, Jiang H, Xu Z, Li H, Wu N, Yin P: Objective secondhand smoke exposure in chronic obstructive pulmonary disease patients without active smoking: the u.s. National health and nutrition examination survey (nhanes) 2007-2012. Ann Transl Med 2020;8:445.

For peer review only

For peer review only - http://bmjopen.bmj.com/site/about/guidelines.xhtml

Table 1. Characteristics of the Study Population in NHANES 2013-2014.

Variable	Overall	Age ≤65 years	Age >65 years
Age ,year	57.38 (56.75-58.01)	51.89 (51.39-52.39)	73.18 (72.72-73.65)
Male ,%	48.08	49.3	44.56
Race/ethnicity, %			
Hispanic-Mexican	6.98	8.03	3.98
Other Ethnicity	11.89	13.0	8.69
Non-Hispanic White	71.01	68.2	79.1
Non-Hispanic Black	10.12	10.78	8.23
Smoking status, %			
Never smoking	53.98	54.38	52.84
Former smoker	28.15	24.27	39.33
Current smoker	17.87	21.35	7.831
Physical activity, %			
Inactive	48.34	45.91	55.31
Moderate	32.75	31.67	35.86
Vigorous	18.91	22.41	8.83
Alcohol intakes, g	4.69 (4.01-5.36)	5.31 (4.45-6.17)	2.98 (2.39-3.56)
BMI, kg/m²	28.54 (28.17-28.9)	28.7 (28.25-29.15)	28.07 (27.56-28.58)
Triglycerides, mmol/L	1.81 (1.73-1.89)	1.84 (1.74-1.94)	1.74 (1.63-1.85)
Total cholesterol, mmol/L	5.09 (5.04-5.13)	5.18 (5.12-5.23)	4.83 (4.70-4.96)
HDL-C, mmol/L	1.42 (1.40-1.43)	1.41 (1.38-1.43)	1.44 (1.39-1.48)
eGFR ,mL/min per 1.73m²	84.28 (83.28-85.28)	89.49 (88.26-90.72)	69.47 (68.52-70.42)
SBP, mmHg	125.42 (124.31-126.53)	122.66 (121.43-123.89)	133.28 (131.63-134.92)
DBP, mmHg	70.75 (69.8-71.71)	72.82 (72.03-73.61)	64.86 (62.81-66.91)
Diabetes, %	14.32	11.39	22.74
Lowering lipid, %	30.02	21.78	53.74
ACEI/ARBs, %	26.87	20.23	45.97
Beta-blockers, %	14.63	8.89	31.14

For peer review only

Statistics of variables were presented as weighted proportions (%) or means (CI%).

Abbreviation: ACEI, angiotensin-converting enzyme inhibitor; ARBs, angiotensin II receptor antagonist; BMI, body mass index; SBP, systolic blood pressure; DBP, diastolic blood pressure; eGFR, estimated glomerular filtration rate; HDL-C, high-density lipoprotein cholesterol.

For peer review only - http://bmjopen.bmj.com/site/about/guidelines.xhtml

For peer review only

For peer review only - http://bmjopen.bmj.com/site/about/guidelines.xhtml

Table 2. Association between age and AAC scores in overall or subgroups by smoking

Models	Overall		Non-smoker		Smoker		p for interaction
**AAC-24**	β coefficient (95%CI)	p value	β coefficient (95%CI)	p value	β coefficient (95%CI)	p value	
crude	0.57 (0.49-0.66)	<0.001	0.49 (0.42-0.57)	<0.001	0.66 (0.53-0.80)	<0.001	0.018
Model 1	0.57 (0.49-0.66)	<0.001	0.50 (0.42-0.58)	<0.001	0.66 (0.52-0.79)	<0.001	0.018
Model 2	0.43 (0.33-0.52)	<0.001	0.37 (0.29-0.44)	<0.001	0.53 (0.38-0.68)	<0.001	0.005
**AAC-8**							
crude	0.20 (0.17-0.23)	<0.001	0.18 (0.15-0.21)	<0.001	0.22 (0.18-0.26)	<0.001	0.081
Model 1	0.20 (0.17-0.23)	<0.001	0.19 (0.16-0.22)	<0.001	0.22 (0.18-0.26)	<0.001	0.081
Model 2	0.16 (0.12-0.19)	<0.001	0.14 (0.11-0.18)	<0.001	0.18 (0.14-0.23)	<0.001	0.017

β coefficient (95%CI) for AAC scores with a 5-year increase of age. p for interaction was estimated by the survey-weighted Wald test was used to assess the potential interaction between age and smoking for AAC.

Model 1 (n=3,140): adjusted for age, sex, and race/ethnicity. Model 2 (n=2,992): additionally adjusted for body mass index (<18.5, 18.5–25, 25–30, or ≥30 kg/m^2), smoking status (adjustment for overall), alcohol consumption(<18.5, 18.5–25, 25–30, or ≥30 kg/m^2), physical activity (less, moderate, vigorous), diabetes, hypertension, TC, TG, HDL-C, eGFR, and lowing lipid agents, ACEI/ARBs, and β-blocker use.

For peer review only - http://bmjopen.bmj.com/site/about/guidelines.xhtml

Figure 1. Spline fitting of aged-related AAC in nonsmokers and smokers

The spline curve shows the relationship between scores of AAC 24 and age in smokers and nonsmokers based on fractional polynomial regression. The solid lines represent point estimates, and dashed lines represent 95% CIs. Non-smokers had a flat trend of age-related calcification before 65 years, whereas the calcification was significantly advanced after 45 years in subjects with smoking.

For peer review only

Figure 2. The visualization of age-related AAC in smokers with quit smoking or not

The spline curve shows the relationship between scores of AAC 24 and age in smokers with quit smoking or not based on fractional polynomial regression. The solid lines represent point estimates.

For peer review only

For peer review only - http://bmjopen.bmj.com/site/about/guidelines.xhtml

Figure 3. Age-related vascular calcification in nonsmokers with or without secondhand smoking exposure

Secondhand smoking exposure was defined by self-report (A), serum cotinine (B), and urinary NNAL levels (C), respectively. The spline curve shows the relationship between scores of AAC 24 and age in nonsmokers with or without SHS exposure based on fractional polynomial regression.

For peer review only

For peer review only - http://bmjopen.bmj.com/site/about/guidelines.xhtml

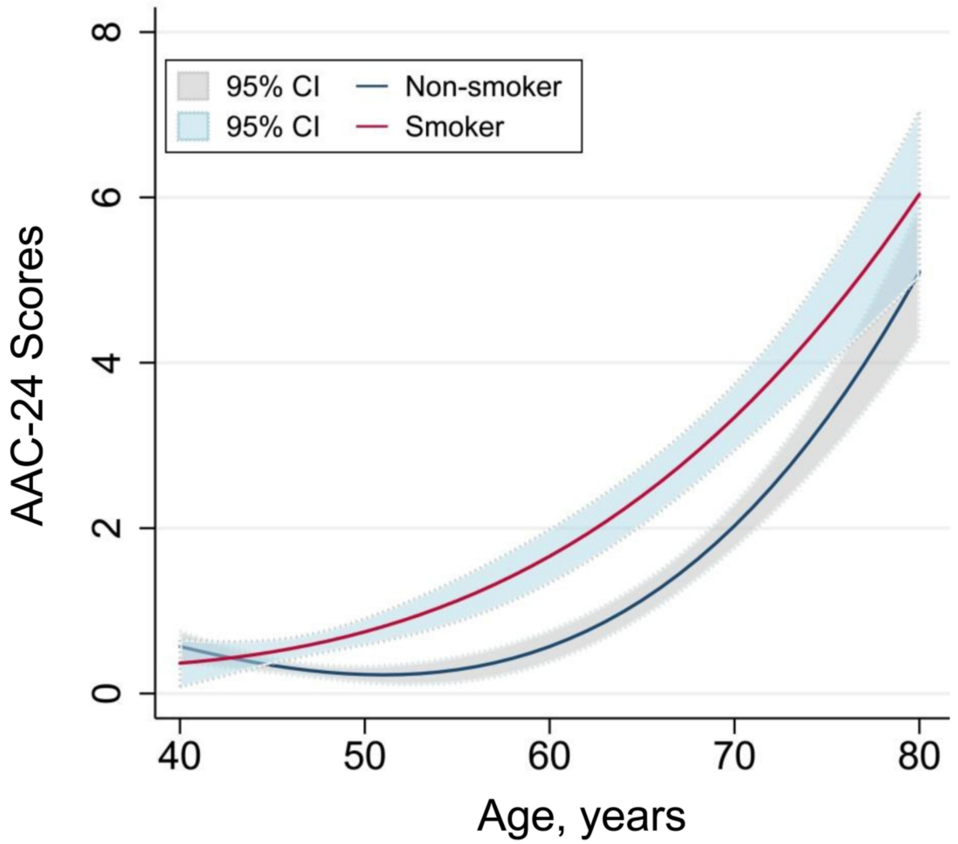

Figure 1. Spline fitting of aged-related AAC in nonsmokers and smokers

112x98mm (600 x 600 DPI)

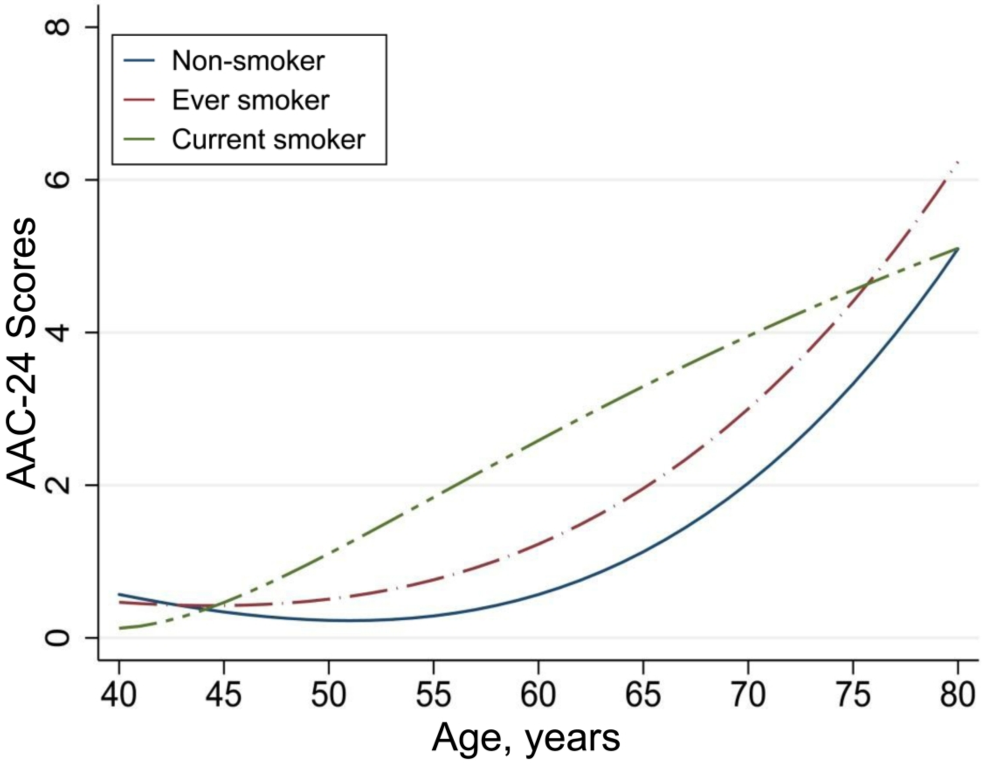

Figure 2. The visualization of age-related AAC in smokers with quit smoking or not

124x96mm (600 x 600 DPI)

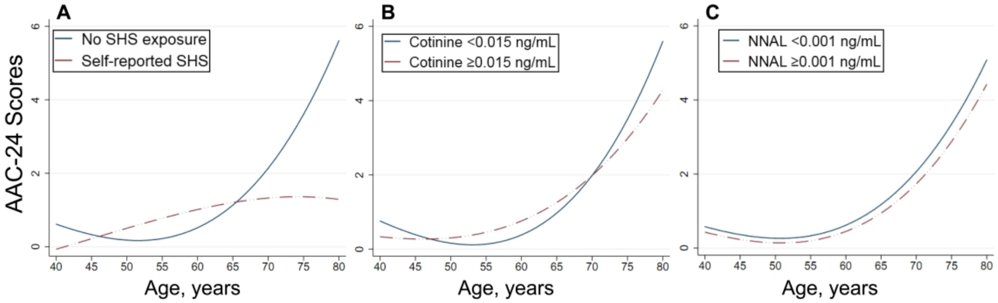

Figure 3. Age-related vascular calcification in nonsmokers with or without secondhand smoking exposure

184x56mm (600 x 600 DPI)

STROBE Statement—Checklist of items that should be included in reports of ***cross-sectional studies***

	Item No	Recommendation	
**Title and abstract**	1	(*a*) Indicate the study's design with a commonly used term in the title or the abstract	P1
		(*b*) Provide in the abstract an informative and balanced summary of what was done and what was found	P2
**Introduction**			
Background/rationale	2	Explain the scientific background and rationale for the investigation being reported	P5
Objectives	3	State specific objectives, including any prespecified hypotheses	P6
**Methods**			
Study design	4	Present key elements of study design early in the paper	P7
Setting	5	Describe the setting, locations, and relevant dates, including periods of recruitment, exposure, follow-up, and data collection	P7-9
Participants	6	(*a*) Give the eligibility criteria, and the sources and methods of selection of participants	P7
Variables	7	Clearly define all outcomes, exposures, predictors, potential confounders, and effect modifiers. Give diagnostic criteria, if applicable	P8-9
Data sources/ measurement	8*	For each variable of interest, give sources of data and details of methods of assessment (measurement). Describe comparability of assessment methods if there is more than one group	P7
Bias	9	Describe any efforts to address potential sources of bias	P10
Study size	10	Explain how the study size was arrived at	P7
Quantitative variables	11	Explain how quantitative variables were handled in the analyses. If applicable, describe which groupings were chosen and why	P9
Statistical methods	12	(*a*) Describe all statistical methods, including those used to control for confounding	P9-10
		(*b*) Describe any methods used to examine subgroups and interactions	P9-10
		(*c*) Explain how missing data were addressed	
		(*d*) If applicable, describe analytical methods taking account of sampling strategy	
		(*e*) Describe any sensitivity analyses	P9-10
**Results**			
Participants	13*	(a) Report numbers of individuals at each stage of study—eg numbers potentially eligible, examined for eligibility, confirmed eligible, included in the study, completing follow-up, and analysed	P11
		(b) Give reasons for non-participation at each stage	
		(c) Consider use of a flow diagram	
Descriptive data	14*	(a) Give characteristics of study participants (eg demographic, clinical, social) and information on exposures and potential confounders	P11
		(b) Indicate number of participants with missing data for each variable of interest	
Outcome data	15*	Report numbers of outcome events or summary measures	P11-12
Main results	16	(*a*) Give unadjusted estimates and, if applicable, confounder-adjusted estimates and their precision (eg, 95% confidence interval). Make clear which confounders were adjusted for and why they were included	

For peer review only

		(*b*) Report category boundaries when continuous variables were categorized	P11-12
		(*c*) If relevant, consider translating estimates of relative risk into absolute risk for a meaningful time period	P11-12
Other analyses	17	Report other analyses done—eg analyses of subgroups and interactions, and sensitivity analyses	
**Discussion**			
Key results	18	Summarise key results with reference to study objectives	P13
Limitations	19	Discuss limitations of the study, taking into account sources of potential bias or imprecision. Discuss both direction and magnitude of any potential bias	P16
Interpretation	20	Give a cautious overall interpretation of results considering objectives, limitations, multiplicity of analyses, results from similar studies, and other relevant evidence	P13-15
Generalisability	21	Discuss the generalisability (external validity) of the study results	P16
**Other information**			
Funding	22	Give the source of funding and the role of the funders for the present study and, if applicable, for the original study on which the present article is based	

*Give information separately for exposed and unexposed groups.

Note: An Explanation and Elaboration article discusses each checklist item and gives methodological background and published examples of transparent reporting. The STROBE checklist is best used in conjunction with this article (freely available on the Web sites of PLoS Medicine at http://www.plosmedicine.org/, Annals of Internal Medicine at http://www.annals.org/, and Epidemiology at http://www.epidem.com/). Information on the STROBE Initiative is available at www.strobe-statement.org.

For peer review only

For peer review only - http://bmjopen.bmj.com/site/about/guidelines.xhtml

BMJ Open

Modified effect of active or passive smoking on the association between age and abdominal aortic calcification: a nationally representative cross-sectional study

Journal:	*BMJ Open*
Manuscript ID	bmjopen-2020-047645.R1
Article Type:	Original research
Date Submitted by the Author:	28-Apr-2021
Complete List of Authors:	Lv, Luyan; Qujing First People's Hospital, Department of Geriatrics, Qujing First People's Hospital<br>Wu, Shixian; Qujing First People's Hospital<br>Yang, Yungui; Qujing First People's Hospital, Department of Geriatrics<br>yue, xiongli; Qujing First People's Hospital, Department of Geriatrics, Qujing First People's Hospital
**Primary Subject Heading**:	Public health
Secondary Subject Heading:	Cardiovascular medicine, Geriatric medicine, Global health, Smoking and tobacco
Keywords:	Cardiology < INTERNAL MEDICINE, Cardiovascular imaging < RADIOLOGY & IMAGING, Vascular medicine < INTERNAL MEDICINE

SCHOLARONE™
Manuscripts

I, the Submitting Author has the right to grant and does grant on behalf of all authors of the Work (as defined in the below author licence), an exclusive licence and/or a non-exclusive licence for contributions from authors who are: i) UK Crown employees; ii) where BMJ has agreed a CC-BY licence shall apply, and/or iii) in accordance with the terms applicable for US Federal Government officers or employees acting as part of their official duties; on a worldwide, perpetual, irrevocable, royalty-free basis to BMJ Publishing Group Ltd ("BMJ") its licensees and where the relevant Journal is co-owned by BMJ to the co-owners of the Journal, to publish the Work in this journal and any other BMJ products and to exploit all rights, as set out in our licence.

The Submitting Author accepts and understands that any supply made under these terms is made by BMJ to the Submitting Author unless you are acting as an employee on behalf of your employer or a postgraduate student of an affiliated institution which is paying any applicable article publishing charge ("APC") for Open Access articles. Where the Submitting Author wishes to make the Work available on an Open Access basis (and intends to pay the relevant APC), the terms of reuse of such Open Access shall be governed by a Creative Commons licence – details of these licences and which Creative Commons licence will apply to this Work are set out in our licence referred to above.

Other than as permitted in any relevant BMJ Author's Self Archiving Policies, I confirm this Work has not been accepted for publication elsewhere, is not being considered for publication elsewhere and does not duplicate material already published. I confirm all authors consent to publication of this Work and authorise the granting of this licence.

For peer review only

Modified effect of active or passive smoking on the association between age and abdominal aortic calcification: a nationally representative cross-sectional study

Brief title: Vascular calcification, age, and smoking

Luyan Lv[1†], Shixian Wu[2†], Yungui Yang[1*], Xiongli Yue[1*]

[1]Department of Geriatrics, Qujing First People's Hospital, Qujing, China;

[2]Ministry of Science and education, Qujing First People's Hospital, Qujing, China;

[†] contributed equally to this article.

Address for correspondence: Xiongli Yue, Yuanlin Road, Qilin District, Qujing, China, 655000, Tel: +86 087 4331 9938, Email: yxl13529891626@163.com;Yungui Yang, Yuanlin Road, Qilin District, Qujing, China, Tel: +86 087 4331 9938, Email: 1369687326@qq.com.

Table number: 2

Figure number: 3

For peer review only

Abstract:

Objectives: Arterial calcification was a critical mark of the atherosclerotic process in aging cardiovascular disease. The deleterious effects of smoking on atherosclerosis were well-known, but the interaction between aging and smoking on atherosclerosis was unclear. this study tested the hypothesis that the association of age with vascular calcification was modified by smoking.

Design: a cross-sectional study

Setting: a nationally representative sample, NHANES 2013–2014 .

Participants: This study incuded 3140 general adults aged 40-80 years with the eligible data of abdominal aortic calcification (AAC). Active and passive smoking exposure was identifed by self-report and tobacco metabolites (serum cotinine and urinary 4-Methylnitrosamino-3-pyridyl-1-butanol).

Primary outcome measures: Abdominal aortic calcification score was determined by dual-energy X-ray absorptiometry (DXA) scans. Multiple regression models were used to assess the association between age, smoking exposure and AAC. Survey-weighted Wald test was used to evaluate the potential interaction.

Results: AAC was positively associated with age in the general population. After adjustment for age, sex, race/ethnicity, and other cardiovascular risk factors, the OR (95CI%) for severe AAC was 1.66 (95CI% 1.48-1.87, p<0.001) per each 5-year increase in age. As expected, the OR for age-related vascular calcification was especially stronger in smokers than never smoker (p for interaction ≤0.014). According to spline fitting, the progression of vascular calcification was significantly

For peer review only - http://bmjopen.bmj.com/site/about/guidelines.xhtml

put forward to 45 years in smokers compared to that after 60 years in never smoker. Quit smoking may compromise the deleteriousness for vascellum especially in younger adults. However, the difference for age-related calcification in never smoker with or without second-hand smoke exposure was minor, regardless of definition by self-report, serum cotinine, or urinary 4-(Methylnitrosamino)-1-(3-pyridyl)-1-butanol.

Conclusions: Smoking significantly accelerates the progression of age-related subclinical atherosclerosis. Early quit smoking should be strengthened among young smokers. The effect of passive smoking exposure on arteriosclerosis should be strictly assessed.

For peer review only

Keywords:

Age, aortic calcification, arteriosclerosis, smoking, second-hand smoking

Strengths and limitations of this study

1. This study was a nationally representative sample of the adult population of US, suggesting a well generalization of the findings.
2. Data was collected with standardized and validated protocols, suggesting a favorable repeatability.
3. This study evaluated the potential modification for age-related abdominal aortic calcification by active smoking or second-hand smoking exposures, defined by self-reports or nicotine metabolites.
4. This study was based on a cross-sectional design that was less likely to conclude the causality.

For peer review only - http://bmjopen.bmj.com/site/about/guidelines.xhtml

5. AAC measurement was only carried out in a population aged 40 years or older. Whether smoking influences age-related vascular calcification in adults less than 40 years old that warrants investigation.

For peer review only

For peer review only - http://bmjopen.bmj.com/site/about/guidelines.xhtml

For peer review only

For peer review only - http://bmjopen.bmj.com/site/about/guidelines.xhtml

Introduction

Cardiovascular disease (CVD) has been the leading cause of death since the early 20th century worldwide, bringing a heavy burden to public health and care[1]. Atherosclerosis has always been the primary pathological basis of CVD among the wide array of underlying cardiovascular lesions[2]. To early identify populations at high risk of subclinical atherosclerosis is essential to prevent the progression of CVD[3]. Abdominal aortic calcification (AAC) was considered as a key hallmark of atherosclerosis and an independent predictor of subsequent cardiovascular morbidity and mortality[1, 4]. Accumulated evidence suggested that severe AAC is associated with a higher risk of major cardiovascular events, reflecting poor cardiovascular health status[5].

Epidemiological studies before decades had demonstrated that vascular calcification or atherosclerosis was a common aging-related process [6, 7]. Along with the improvement of the primary and second prevention strategies for cardiovascular events, how the association between age and aortic calcification during the contemporary era is unknown. Smoking is a well-established modifiable risk factor for atherosclerosis and CVD via several underlying pathways[8], and has been a critical environmental hazard for public health[9]. It is noteworthy that, approximately 11% of cardiovascular death is attributed to smoking exposure worldwide[10]. Furthermore, secondhand smoke (SHS) exposure in non-smoker was also associated with cardiovascular disease and other health consequences[11]. Although numerous studies demonstrated the associations between ageing or smoking

For peer review only - http://bmjopen.bmj.com/site/about/guidelines.xhtml

and atherosclerotic events, the evidence on the interaction effect between ageing and active or passive smoking in aortic calcification was limited. To clarify this issue may provide new insights into the atherosclerosis process and identify the targeted population benefited from smoking cessation education, . In this context, this study tested the hypothesis that the association between age and abdominal aortic calcification was modified by smoking exposure, including active and secondhand smokine, in a nationally representative sample of the middle-aged and elderly population.

For peer review only - http://bmjopen.bmj.com/site/about/guidelines.xhtml

Methods

Study Population

The data were extracted from National Health and Nutrition Examination Surveys (NHANES) 2013-2014, an ongoing nationally representative, stratified, multistage probability-sampling survey designed by the Center for Disease Control and Prevention of the United States. The methods and protocols had been described in previous literatures[12, 13]. There were 10,175 individuals in NHANES 2013-2014 and 3,815 adults 40 years of age and older.. Pregnant females were excluded (n=3). dual-energy X-ray absorptiometry (DXA) scans were conducted in participants aged 40 years and older. Aortic calcification adjacent to lumbar vertebrae L1–L4 was assessed. Exluding participants without scan (n=482) or image invalidity (n=190). All those with eligible AAC scores were included in this study (n = 3140). This study was conducted in accordance with the Declaration of Helsinki. The protocols of NHANES were approved by the National Center for Health Statistics ethics review board. Informed consent was obtained from all participants. Detailed information is available on the website http://www.cdc.gov/nchs/nhanes/irba98.htm.

Abdominal Aortic Calcification Measurement

Abdominal aortic calcification (AAC) could be easily assessed on lateral spine scans obtained by DXA with some advantages including inexpensive, ease, rapid and safe. The scores of AAC were calculated from a lateral scan of the lumbar spine (vertebrae L1-L4) that was acquired on Hologic Discovery model A densitometers

For peer review only

For peer review only - http://bmjopen.bmj.com/site/about/guidelines.xhtml

(Hologic, Inc., Marlborough, Massachusetts), using software Apex 3.2 version[1, 14]. The radiation exposure from dual-energy X-ray absorptiometry (DXA) for the lateral spine scan was extremely low (less than 20 uSv). All scans were viewed using Optasia SpinAnalyzer software. The 24-point semi-quantitative score (AAC-24) (Kauppila score) semi-quantitative score was applied for the primary analysis, and AAC-8 score (Schousboe score) was used for sensitive analysis. Both methods had been validated with favorable sensitivity and specificity to reflect the severity of abdominal aortic calcification. AAC-24 was calculated according to the length of calcification at the posterior and anterior aortic walls contiguous to lumbar spine L1-L4. Treating lines across the middle of intervertebral spaces as segment boundaries, abdominal aorta was divided into 8 segments. AAC was scored from 0 to 3 according to calcification length in aortic wall of each segment (0 point: no calcification; 1 point: ≤1/3 arterial wall in each segment; 2 points: 1/3-2/3; 3 points: >2/3). The AAC-8 was a simplified method derived from AAC-24. AAC-8 in anterior or posterior aortic walls in front of L1-L4 was scored 0 to 4, respectively (0 point: no calcification; 1 point: no more than the length of one vertebrae; 2 points: no more than the length of 2 vertebra; 3 points: no more than the length of 3 vertebra; 4 points: more than the length of three vertebra). Therefore, AAC-8 score was less influenced by small calcification but needed more skillful technologists than AAC-24. The DXA examinations were administered by trained and certified radiology technologists. The detailed protocols have been presented in previous studies[1, 14].

For peer review only - http://bmjopen.bmj.com/site/about/guidelines.xhtml

Demographic, clinical, and laboratory variables

Data concerning age, sex, race/ethnicity, smoking status, physical activity, and alcohol consumptions, history of the disease was collected during personal interviews using standardized questionnaires[12]. Physical examination was conducted in the Mobile Examination Centre (MEC) with standardized protocols. Peripheral blood was commenced were collected in MEC and determined in the central laboratory following the standardized procedures. Race/ethnicity was categorized into 4 groups: non-Hispanic white, non-Hispanic black, Hispanic-Mexican, and others. Physical activity was categorized as vigorous, moderate, and less activity as presented elsewhere. Drinking was assessed via questionnaires for the preceding year. One drink was defined as 12-oz beer, 4-oz wine, or 1 ounce of liquor, each covering about 10 g of alcohols[15]. Body mass index (BMI) was presented as weight divided by height squared (kg/m2). Triglyceride (TG), total cholesterol (TC), high-density lipoprotein-cholesterol (HDL-C), and serum creatinine were determined in subjects with morning fasting status. The estimated glomerular filtration rate (eGFR) was calculated by the Chronic Kidney Disease Epidemiology Collaboration (CKD-EPI). Hypertension was defined as antihypertensive administration, systolic BP ≥140 mmHg, or diastolic BP ≥90 mmHg. Diabetes was defined as anti-hyperglycemic therapy or HbA1c ≥6.5%[15].

Smoking was defined as smoke at least 100 cigarettes during his or her life, ascertained through self-reports including both past and current cigarette smoking status. Tobacco metabolites, serum cotinine, and urinary 4-(Methylnitrosamino)-1-

For peer review only - http://bmjopen.bmj.com/site/about/guidelines.xhtml

(3-pyridyl)-1-butanol (NNAL) were considered as biomarkers of passive smoking exposures in non-smoker [16]. Three definitions were used to identify individuals with SHS exposure, including self-report, urinary NNAL≥0.001ng/mL, or serum cotinine ≥0.015ng/mL, as previous mentioned. Serum cotinine and urinary NNAL were determined by high-performance liquid chromatography-tandem mass spectrometric (HPLC-MS) method, with atmospheric pressure chemical ionization or electrospray ionization, respectively. Biosample was pretreated with methyl-D3-cotinine or 4-(Methylnitrosoamino)-1-(3-pyridyl)-1-butanol-1,2',3',4',5',6'-$13C^6$ as the internal standard. C18 HPLC column was used to separate the samples. The eluent from these injections was monitored by a mass spectrometer. The m/z 80 product ion from the m/z 177 quasi-molecular ion was used to quantify serum cotinine.

Statistical Analysis

Continuous and categorical variables are expressed as mean (SE) and percentage, weighted with primary sampling unit, pseudo-strata, and appropriate sampling weights to account for the complex sampling design. Crude and multivariable logistics or linear regression models were applied to assess the association of age with scores of abdominal aortic calcification. According to previous studies[1, 14], severe AAC was defined as AAC-24 ≥6 points and subclinical AAC was defined as AAC-24 ≥2 points. Logistics regression was applied to calculate odds ratios as primary analysis. Two adjusted models were adopted. Model 1 was adjusted for sex, and race/ethnicity. Model 2 was additionally adjusted for body mass index, smoking

For peer review only - http://bmjopen.bmj.com/site/about/guidelines.xhtml

status, alcohol consumption, physical activity, diabetes, hypertension, total cholesterol, HDL-C, estimated glomerular filtration rate, and lowing lipid agents. VIF of each variable was not more than 2.64 that did not suggest a multicollinearity of covariates. The survey-weighted Wald test was used to assess the potential interaction between age and smoking for AAC. The β coefficient (95%CI) was presented as the increased scores of AAC-24 or AAC-8 for each 5-year increase. Two-way fractional polynomial regression spline plots were used to visualize the relationship between age and vascular calcification among smokers or never smokers, and SHS or non-SHS. Statistical analysis was conducted with Stata (version SE15.0). All tests with a 2-sided $P<0.05$ were considered statistically significant.

For peer review only

Results

Overall, 3140 adults aged ≥40 years had measured abdominal aortic calcification in NHANES 2013-2014 (**Table 1**). The weighted mean age was 57.4 years and 51.9% (n= 1622) were female. There are 54.0% never smokers and 46.0% smokers, including 28.2% ever smoking and 17.8% current smoking, according to self-report. Compared to those age ≤ 65 years, elder participants had higher proportions of non-Hispanic whites, quit smoking, diabetes, and cardiovascular medications, as well as less physical activity and lower renal function.

The association between age and abdominal aortic calcification in all participants or stratified by smoking was shown in **Table 2 and Table S1**. In univariable and sex- and race-adjusted logistics regression models, age was significantly associated with severe or subclinical ACC, with a 85% and 44% increased risk for each 5-year increase in age(each p<0.001). After adjustment for sex, race, smoking, physical activity, alcohol intakes, BMI, hypertension, diabetes, TC, TG, HDL-C, eGFR, and lowering-lipid agents, the association of age and AAC remained significant with OR (95%CI) 1.66 (1.48-1.87) and 1.37 (1.25-1.50) for each 5-year increase in age (each p<0.001) , respectively (**Table 2)**. In addition, AAC-24 and AAC-8 scores were also positively associated with age **(Table S1)**. The association of age and AAC-24 remained significant (β coefficient: 0.43 95CI% 0.33-0.52 for each 5-year increase in age, p<0.001). Supplementary analysis for the association between smoking exposure and AAC was conducted (**Table S2-4**). As aspected, current smoking was significantly associated with AAC severity which was weakened in participants with

For peer review only - http://bmjopen.bmj.com/site/about/guidelines.xhtml

smoking cessation more than 10 years. However, the relationship between passive smoking and AAC was not noted.

Because smoking cigarettes was a strong risk factor for vascular injury, we assessed age-related aortic calcification in subgroups by smoking status (**Table 2 and Table S1**). As expected, the effect modification by smoking was observed (p for interaction ≤0.014). In fully adjusted models, the OR (95%CIs) per each 5-year increase of age for severe AAC were 1.67 (1.50-1.85) among participants without smoking versus 2.03 (1.70-2.42) among participants with smoking. That suggested that ever and current smoking may quicken age-related abdominal aortic calcification by 30% compared to that in never smokers. The repeated analysis was performed using binary subclinical AAC, AAC-24 ad AAC-8 scores, and the correlations were unchanged. According to the fitting splines (**Figure 1**), never smokers had a flat trend of age-related calcification before 65 years, whereas the calcification was significantly advanced after 45 years in subjects with smoking. Furthermore, compared to current smokers, participants with quit smoking had lower scores of age-related calcification, especially for those aged 50-70 years (**Figure 2**). However, among never smokers, secondhand smoking (SHS) exposure did not significantly affect age-related abdominal aortic calcification (**Figure 3**).

For peer review only - http://bmjopen.bmj.com/site/about/guidelines.xhtml

Discussion

In this nationally representative sample from the US general population, the association between abdominal aortic calcification and age differed depending on smoking status after adjustment for various confounders. Age-related calcification was flat before 60 years, while that was driven by smoking since 45 years. Quit smoking may relatively mitigate the process of age-induced calcification compared to persistent smokers, especially for the population aged 45-70 years. However, the deleterious effect of secondhand smoking on vascular calcification in adults was not observed. Our study is of particular interest because our findings highlight the benefits of never smoking and early quit smoking education on the process of age-related vascular calcification or atherosclerosis in the contemporary population.

Vascular calcification was considered to be a degenerative and end-stage consequence of aging processes[6]. Arterial calcification was detected in approximately one-fourth in populations at 50 years old, and the proportion subsequently increased to beyond 60% in population aged more than 75 years[7]. Consistently, our results also noted that the scores of AAC were slowly increased in middle-aged persons, and robustly increased in elderly aged $\geq$ 65 years.

Vascular calcification has been linked with vascular injury and subclinical atherosclerotic disease[14, 17]. Vascular wall calcification was a common byproduct of atherosclerotic plaques and thus was considered as a potential marker of subclinical atherosclerosis as well as a simple tool for cardiovascular risk stratification[14]. Although previous reports have highlighted the implications of AAC as a biomarker

For peer review only - http://bmjopen.bmj.com/site/about/guidelines.xhtml

of cardiovascular morbidity and mortality in general adults[18-20], AAC as a modifiable marker in preventing atherosclerosis and cardiovascular risk has received less attention[21]. Our findings suggested quit smoke is a simple and effective means to mitigate the process of arterial calcification and atherosclerosis in the general population, especially in young adults. The change of lifestyle seems to be promising, low-cost, and easy to spread.

Hisamatsu et al. conducted an interesting study and demonstrated a positive association between smoking and subclinical atherosclerosis assessed by coronary artery calcification, carotid intima-media thickness and carotid plaque, aortic artery calcification (from aortic arch to iliac bifurcation), and ankle-brachial index comprehensively[22]. The relationship attenuated with the time of smoking cessation Moreover, Pham et al. further supplemented the analysis for the relationship between smoking habits and progression of coronary and aortic artery calcification in a follow-up study[23]. Those findings supported that smoking is a strong risk factors of coronary calcification and atherosclerosis [24-26]. Jung and colleagues observed the positive association between AAC and long-term smoking exposure in 218 men aged 40 to 81 years. Even a low dosage of smoking also increased the risk of AAC[27]. Furthermore, Hirooka and colleagues analyzed 313 Japanese and 302 Korean men aged 40-49 years and concluded that smoking with higher pack-years was significantly associated with artery calcium, suggesting a dose-response pattern between cigarette use and progression of atherosclerosis[24]. A small sample size and selection bias may limit the extrapolation. Our analysis also supported the increased

For peer review only - http://bmjopen.bmj.com/site/about/guidelines.xhtml

risk of severe or subclinical angiosteosis associated with smoking. Notably, smoking cessation more than 10 years could partly attenuate the harm of smoke exposure that was consistent in previous reports[22, 23, 28]. And our results further emphasized that the association between age and ACC was modified by smoking, and the detrimental effects of ageing and smoking were not simply superposed. .

Based on NHANES data, one recent study noted that serum cotinine levels, a biomarker of smoking, were associated with the risk of severe abdominal aortic calcification in 2840 adults[14]. However, circulating cotinine only reflected short-term exposure of smoking, and the interaction between smoking and age on the process of vascular calcification was unknown. Our results highlighted that the process of age-related calcification was slow before 60 years old. By contrast, smoking, especially persistent smoking significantly accelerated this process since 40-years old. Early quit smoking may at least part moderate the speed for adults aged ≤70 years. This is the first study that specifically investigates the interaction between age and smoking on the severity of aortic calcification in humans. Notably, previous studies have been established the association between smoking and vascular calcification, suggesting the potential benefits of quit smoking to prevent atherosclerosis[8, 14, 25, 26]. However, the complex interaction with the aging process may be neglected that may provide new insights into the optimal time of preventing atherosclerosis by tobacco control[26]. Our results suggested quit smoking may have little advantage in elderly aged >70 years that highlights the importance of early action to reduce the burden of vascular calcification.

For peer review only - http://bmjopen.bmj.com/site/about/guidelines.xhtml

Secondhand smoke (SHS) exposure has been proved to be associated with cardiovascular disease and adverse outcomes[11]. About 85% of SHS was derived from side-stream smoke that seemed potentially more harmful due to non-filtering[29]. As another purpose, we investigated the relationship between AAC and SHS with the definition by self-report, serum cotinine or urine 4-(methylnitrosamino)-1-(3-pyridyl)-1-butanonol (NNAL) in never smokers. However, the neutral link between arterial calcification and passive smoking we noted might be not the ultimate conclusion. The questionnaires of secondhand smoking were only designed for the preceding 7 days, although we used the definition of potentially persistent SHS exposure indoors at job or at home. Serum cotinine and urinary NNAL, as more accurate biomarkers of smoking exposure than self-reported, had been validated to estimate the SHS exposure of the past 2–4 days and 6–12 weeks, respectively[30]. The relationship was also insignificant in stratifications by cotinine or NNAL. The serum cotinine or urine NNAL only reflected exposures over the past several days or weeks that was insufficient to reflect the long-term exposure. The progression of vascular calcification was a chronic process even beyond the years. Thus, further study may need multiple repeated measurements or questionnaires over years to assess the effect of SHS on age-related calcification.

Limitations and strengths

Our study has some strengths that support a favorable repeatability, including nationally representative of the adult population, standardized and validated protocols, full adjustment for a large number of potential confounders. However, our

For peer review only - http://bmjopen.bmj.com/site/about/guidelines.xhtml

conclusions should be explained in consideration of the following limitations. First, this study was based on a cross-sectional design that was less likely to conclude the causality. More convincing evidence could be obtained from a longitudinal study with multiple measurements. Second, the analysis for the relationship between secondhand smoking and AAC was not the ultimate seal. The duration of SHS should be considered in further study. Third, AAC measurement was only carried out in a population aged 40 years or older. Whether smoking influences age-related vascular calcification in adults less than 40 years old that warrants investigation. Forth, residual confounding could not be eliminated, though we strictly adjusted for 16 covariates in the regression model.

Conclusions

Our results indicate that smoking significantly modifies the association between age and the severity of abdominal aortic calcification in adults aged 40 years or older. Age-related vascular calcification is accelerated by smoking that is partly reduced by quit smoking. However, whether secondhand smoking affects this process needs further investigation.

For peer review only - http://bmjopen.bmj.com/site/about/guidelines.xhtml

Acknowledgments: The authors would like to thank all participants and staff in NHANES for their great contributions.

Data availability statement: All data are publicly available on the website http://www.cdc.gov/nchs/nhanes/irba98.htm.

Ethics Approval: The protocols and procedures of this study were agreed by the Research Ethics Review Board of the Centers of Disease Control and Prevention of United States (Protocol Number. 2011-17).

Conflict of interest: The authors declare no potential conflict of interest.

Sources of Funding: This manuscript was written independently. The authors received no financial support.

Author contributions:

Yungui Yang conceived the design of the study. Yungui Yang and Xiongli Yue developed the protocols. Luyan Lv and Shixian Wu organized and analyzed all data. All authors wrote and reviewed the manuscript. Yungui Yang and Xiongli Yue take the responsibility for the integrity and accuracy of data.

Patient and public involvement

No patients were involved in the development of the research question or design of the study.

References:

[1]. Li, Y.W. and W.L. Chen, Clinical relevance of serum uric acid and abdominal aortic-calcification in a national survey. Clin Cardiol, 2020.

[2]. Lusis, A.J., Atherosclerosis. Nature, 2000. 407(6801): p. 233-41.

[3]. Kalsch, H., et al., Aortic Calcification Onset and Progression: Association With the Development of Coronary Atherosclerosis. J Am Heart Assoc, 2017. 6(4).

For peer review only - http://bmjopen.bmj.com/site/about/guidelines.xhtml

[4]. Wilson, P.W., et al., Abdominal aortic calcific deposits are an important predictor of vascular morbidity and mortality. Circulation, 2001. 103(11): p. 1529-34.
[5]. Szulc, P., Abdominal aortic calcification: A reappraisal of epidemiological and pathophysiological data. Bone, 2016. 84: p. 25-37.
[6]. Zhang, B., V.M. Miller and J.D. Miller, Influences of Sex and Estrogen in Arterial and Valvular Calcification. Front Endocrinol (Lausanne), 2019. 10: p. 622.
[7]. Cannata-Andia, J.B., et al., Vascular calcifications: pathogenesis, management, and impact on clinical outcomes. J Am Soc Nephrol, 2006. 17(12 Suppl 3): p. S267-73.
[8]. Wang, Z., D. Wang and Y. Wang, Cigarette Smoking and Adipose Tissue: The Emerging Role in Progression of Atherosclerosis. Mediators Inflamm, 2017. 2017: p. 3102737.
[9]. Smoking prevalence and attributable disease burden in 195 countries and territories, 1990-2015: a systematic analysis from the Global Burden of Disease Study 2015. Lancet, 2017. 389(10082): p. 1885-1906.
[10]. Ezzati, M., et al., Role of smoking in global and regional cardiovascular mortality. Circulation, 2005. 112(4): p. 489-97.
[11]. Asfar, T., et al., Secondhand Smoke Exposure Among High-Risk Patients in the United States (NHANES 2001-2012): Implications for Clinical Practice. Nicotine Tob Res, 2019. 21(4): p. 551-556.
[12]. Wang, S., et al., Mitochondria-derived methylmalonic acid, a surrogate biomarker of mitochondrial dysfunction and oxidative stress, predicts all-cause and cardiovascular mortality in the general population. Redox Biol, 2020. 37: p. 101741.
[13]. Rossato, L.T., et al., Association between omega-3 fatty acids intake and muscle strength in older adults: A study from National Health and Nutrition Examination Survey (NHANES) 1999-2002. Clin Nutr, 2020.
[14]. Wen, X., et al., Association Between Serum Cotinine and Severe Abdominal Aortic Calcification in US Adults. Angiology, 2020. 71(4): p. 333-339.
[15]. Wang, S., et al., Temporal trend of circulating trans-fatty acids and risk of long-term mortality in general population. Clin Nutr, 2020.
[16]. Fu, Z., et al., Objective secondhand smoke exposure in chronic obstructive pulmonary disease patients without active smoking: the U.S. National Health and Nutrition Examination Survey (NHANES) 2007-2012. Ann Transl Med, 2020. 8(7): p. 445.
[17]. Budoff, M.J., et al., Assessment of coronary artery disease by cardiac computed tomography: a scientific statement from the American Heart Association Committee on Cardiovascular Imaging and Intervention, Council on Cardiovascular Radiology and Intervention, and Committee on Cardiac Imaging, Council on Clinical Cardiology. Circulation, 2006. 114(16): p. 1761-91.
[18]. Bastos, G.F., et al., Calcification of the abdominal aorta as an independent predictor of cardiovascular events: a meta-analysis. Heart, 2012. 98(13): p. 988-94.
[19]. Martino, F., et al., Abdominal aortic calcification is an independent predictor of cardiovascular events in peritoneal dialysis patients. Ther Apher Dial, 2013. 17(4): p. 448-53.
[20]. Bolland, M.J., et al., Abdominal aortic calcification on vertebral morphometry images predicts incident myocardial infarction. J Bone Miner Res, 2010. 25(3): p. 505-12.
[21]. Golledge, J., Abdominal aortic calcification: clinical significance, mechanisms and therapies. Curr Pharm Des, 2014. 20(37): p. 5834-8.
[22]. Hisamatsu, T., et al., Smoking, Smoking Cessation, and Measures of Subclinical Atherosclerosis in Multiple Vascular Beds in Japanese Men. J Am Heart Assoc, 2016. 5(9).

For peer review only - http://bmjopen.bmj.com/site/about/guidelines.xhtml

[23]. Kianoush, S., et al., Associations of Cigarette Smoking With Subclinical Inflammation and Atherosclerosis: ELSA-Brasil (The Brazilian Longitudinal Study of Adult Health). J Am Heart Assoc, 2017. 6(6).
[24]. Hirooka, N., et al., Influence of cigarette smoking on coronary artery and aortic calcium among random samples from populations of middle-aged Japanese and Korean men. J Epidemiol Community Health, 2013. 67(2): p. 119-24.
[25]. Rasmussen, T., et al., Development and progression of coronary artery calcification in long-term smokers: adverse effects of continued smoking. J Am Coll Cardiol, 2013. 62(3): p. 255-257.
[26]. Lehmann, N., et al., Effect of smoking and other traditional risk factors on the onset of coronary artery calcification: results of the Heinz Nixdorf recall study. Atherosclerosis, 2014. 232(2): p. 339-45.
[27]. Jung, J.G., et al., Relationship between Smoking and Abdominal Aorta Calcification on Computed Tomography. Korean J Fam Med, 2019. 40(4): p. 248-253.
[28]. McEvoy, J.W., et al., Relationship of cigarette smoking with inflammation and subclinical vascular disease: the Multi-Ethnic Study of Atherosclerosis. Arterioscler Thromb Vasc Biol, 2015. 35(4): p. 1002-10.
[29]. Park, Y.S., et al., Association between secondhand smoke exposure and hypertension in never smokers: a cross-sectional survey using data from Korean National Health and Nutritional Examination Survey V, 2010-2012. BMJ Open, 2018. 8(5): p. e021217.
[30]. Fu, Z., et al., Objective secondhand smoke exposure in chronic obstructive pulmonary disease patients without active smoking: the U.S. National Health and Nutrition Examination Survey (NHANES) 2007-2012. Ann Transl Med, 2020. 8(7): p. 445.

For peer review only - http://bmjopen.bmj.com/site/about/guidelines.xhtml

Tables and figure legends

Table 1. Characteristics of the Study Population in NHANES 2013-2014.

Variable	Overall	Age ≤65 years	Age >65 years
Age ,year	57.38 (56.75-58.01)	51.89 (51.39-52.39)	73.18 (72.72-73.65)
Male ,%	48.08	49.3	44.56
Race/ethnicity, %			
Hispanic-Mexican	6.98	8.03	3.98
Other Ethnicity	11.89	13.0	8.69
Non-Hispanic White	71.01	68.2	79.1
Non-Hispanic Black	10.12	10.78	8.23
Smoking status, %			
Never smoking	53.98	54.38	52.84
Former smoker	28.15	24.27	39.33
Current smoker	17.87	21.35	7.831
Physical activity, %			
Inactive	48.34	45.91	55.31
Moderate	32.75	31.67	35.86
Vigorous	18.91	22.41	8.83
Alcohol intakes, g	4.69 (4.01-5.36)	5.31 (4.45-6.17)	2.98 (2.39-3.56)
BMI, kg/m^2	28.54 (28.17-28.9)	28.7 (28.25-29.15)	28.07 (27.56-28.58)
Triglycerides, mmol/L	1.81 (1.73-1.89)	1.84 (1.74-1.94)	1.74 (1.63-1.85)
Total cholesterol, mmol/L	5.09 (5.04-5.13)	5.18 (5.12-5.23)	4.83 (4.70-4.96)
HDL-C, mmol/L	1.42 (1.40-1.43)	1.41 (1.38-1.43)	1.44 (1.39-1.48)
eGFR ,mL/min per 1.73m^2	84.28 (83.28-85.28)	89.49 (88.26-90.72)	69.47 (68.52-70.42)
SBP, mmHg	125.42 (124.31-126.53)	122.66 (121.43-123.89)	133.28 (131.63-134.92)
DBP, mmHg	70.75 (69.8-71.71)	72.82 (72.03-73.61)	64.86 (62.81-66.91)
Diabetes, %	14.32	11.39	22.74
Lowering lipid, %	30.02	21.78	53.74
ACEI/ARBs, %	26.87	20.23	45.97
Beta-blockers, %	14.63	8.89	31.14

Statistics of variables were presented as weighted proportions (%) or means (CI%).

Abbreviation: ACEI, angiotensin-converting enzyme inhibitor; ARBs, angiotensin II receptor antagonist; BMI, body mass index; SBP, systolic blood pressure; DBP, diastolic blood pressure; eGFR, estimated glomerular filtration rate; HDL-C, high-density lipoprotein cholesterol.

For peer review only - http://bmjopen.bmj.com/site/about/guidelines.xhtml

Table 2. Association between age and severe or subclinical AAC in overall or subgroups by smoking

Models	Overall		Never smoker		Smoker		p for interaction
**Severe AAC**	**OR (95%CI)**	p value	**OR (95%CI)**	p value	**OR (95%CI)**	p value	
Crude	1.84 (1.68-2.02)	<0.001	1.67 (1.50-1.85)	<0.001	2.03 (1.70-2.42)	<0.001	0.008
Model 1	1.85 (1.69-2.02)	<0.001	1.67 (1.50-1.85)	<0.001	2.07 (1.74-2.46)	<0.001	0.008
Model 2	1.66 (1.48-1.87)	<0.001	1.55 (1.32-1.82)	<0.001	1.78 (1.52-2.09)	<0.001	0.014
**Subclinical AAC**							
Crude	1.44 (1.32-1.56)	<0.001	1.29 (1.17-1.44)	<0.001	1.55 (1.43-1.68)	<0.001	0.002
Model 1	1.44 (1.32-1.57)	<0.001	1.29 (1.16-1.43)	<0.001	1.55 (1.45-1.68)	<0.001	0.001
Model 2	1.37 (1.25-1.50)	<0.001	1.26 (1.14-1.39)	<0.001	1.45 (1.31-1.61)	<0.001	0.011

Severe AAC was defined as AAC-24 ≥6 points and subclinical AAC was defined as AAC-24 ≥2 points. OR (95%CI) for the risk of severe AAC or subclinical AAC was estimated by weighted logistics regression analysis. p for interaction was used to assess the potential interaction between age and smoking for AAC by the survey-weighted Wald test. Model 1 (n=3,140): adjusted for sex, and race/ethnicity. Model 2 (n=2,992): additionally adjusted for body mass index, smoking status, alcohol consumption, physical activity, diabetes, hypertension, TC, TG, HDL-C, eGFR, and lowing lipid agents.

For peer review only - http://bmjopen.bmj.com/site/about/guidelines.xhtml

Figure 1. Spline fitting of aged-related AAC in never smokers and smokers

The spline curve shows the relationship between scores of AAC-24 and age in smokers and nonsmokers based on fractional polynomial regression. The solid lines represent point estimates, and dashed lines represent 95% CIs. AAC-24 was calculated according to the length of calcification at the posterior and anterior aortic walls contiguous to lumbar spine L1-L4. Treating lines across the middle of intervertebral spaces as segment boundaries, abdominal aorta was divided into 8 segments. AAC was scored from 0 to 3 according to calcification length in aortic wall of each segment (0 point: no calcification; 1 point: ≤1/3 arterial wall in each segment; 2 points: 1/3-2/3; 3 points: >2/3). Never smokers had a flat trend of age-related calcification before 65 years, whereas the calcification was significantly advanced after 45 years in subjects with smoking.

For peer review only

Figure 2. The visualization of age-related AAC in smokers with quit smoking or not

The spline curve shows the relationship between scores of AAC-24 and age in smokers with quit smoking or not based on fractional polynomial regression. The solid lines represent point estimates.

For peer review only

For peer review only - http://bmjopen.bmj.com/site/about/guidelines.xhtml

Figure 3. Age-related vascular calcification in never smoker with or without secondhand smoking exposure

Secondhand smoking exposure was defined by self-report (A), serum cotinine (B), and urinary NNAL levels (C), respectively. The spline curve shows the relationship between scores of AAC-24 and age in nonsmokers with or without SHS exposure based on fractional polynomial regression.

For peer review only

For peer review only - http://bmjopen.bmj.com/site/about/guidelines.xhtml

Supplemental data

Table S1. Association between age and AAC scores in overall or subgroups by smoking

Models	Overall		Never smoker		Smoker		p for interaction
**AAC-24**	β coefficient (95%CI)	p value	β coefficient (95%CI)	p value	β coefficient (95%CI)	p value	
Crude	0.57 (0.49-0.66)	<0.001	0.49 (0.42-0.57)	<0.001	0.66 (0.53-0.80)	<0.001	0.018
Model 1	0.57 (0.49-0.66)	<0.001	0.50 (0.42-0.58)	<0.001	0.66 (0.52-0.79)	<0.001	0.018
Model 2	0.43 (0.33-0.52)	<0.001	0.37 (0.29-0.44)	<0.001	0.53 (0.38-0.68)	<0.001	0.005
**AAC-8**							
Crude	0.20 (0.17-0.23)	<0.001	0.18 (0.15-0.21)	<0.001	0.22 (0.18-0.26)	<0.001	0.081
Model 1	0.20 (0.17-0.23)	<0.001	0.19 (0.16-0.22)	<0.001	0.22 (0.18-0.26)	<0.001	0.081
Model 2	0.16 (0.12-0.19)	<0.001	0.14 (0.11-0.18)	<0.001	0.18 (0.14-0.23)	<0.001	0.017

β coefficient (95%CI) for AAC scores with a 5-year increase of age. p for interaction was estimated by the survey-weighted Wald test was used to assess the potential interaction between age and smoking for AAC. Model 1 (n=3,140): adjusted for sex, and race/ethnicity. Model 2 (n=2,992): additionally adjusted for body mass index, smoking status, alcohol consumption, physical activity, diabetes, hypertension, TC, TG, HDL-C, eGFR, and lowing lipid agents.

For peer review only - http://bmjopen.bmj.com/site/about/guidelines.xhtml

Table S2. Association between active smoking status and AAC scores

	Never smoking	Quit smoking	Current smoking	
**AAC-8**		**β (95%CI)**	**β (95%CI)**	**p for trend**
Crude	Reference	0.31 (0.17-0.45)	0.23 (0.04-0.43)	0.002
Model 1	Reference	0.15 (0.01-0.29)	0.40 (0.22-0.58)	<0.001
Model 2	Reference	0.14 (0.00-0.29)	0.29 (0.12-0.46)	0.003
**AAC-24**		**β (95%CI)**	**β (95%CI)**	
Crude	Reference	0.92 (0.55-1.29)	0.51 (0.08-0.94)	0.001
Model 1	Reference	0.46 (0.12-0.79)	0.96 (0.54-1.38)	<0.001
Model 2	Reference	0.46 (0.10-0.81)	0.70 (0.34-1.05)	0.001
**Severe AAC**		**OR (95%CI)**	**OR (95%CI)**	
Crude	Reference	2.02 (1.51-2.71)	1.59 (0.94-2.68)	0.012
Model 1	Reference	1.66 (1.18-2.35)	4.30 (2.40-7.71)	<0.001
Model 2	Reference	1.69 (1.12-2.56)	3.39 (2.04-5.66)	<0.001
**Subclinical AAC**		**OR (95%CI)**	**OR (95%CI)**	
Crude	Reference	1.85 (1.37-2.49)	1.73 (1.12-2.66)	0.003
Model 1	Reference	1.47 (1.05-2.07)	2.78 (1.82-4.25)	<0.001
Model 2	Reference	1.46 (1.00-2.14)	2.06 (1.34-3.16)	0.004

β coefficient (95%CI) for AAC scores acorss the smoking status with never smoking as reference. Model 1 (n=3,140): adjusted for age, sex, and race/ethnicity. Model 2 (n=2,992): additionally adjusted for body mass index, alcohol consumption, physical activity, diabetes, hypertension, TC, TG, HDL-C, eGFR, and lowing lipid agents.

For peer review only - http://bmjopen.bmj.com/site/about/guidelines.xhtml

Table S3. Association between smoking cessation and AAC scores in smokers

	Current smoking	Smoking Cessation <10y	Smoking Cessation ≥10y	p for trend
**AAC-24**				
**Subclinical AAC**	Reference	0.97 (0.63-1.48)	0.65 (0.44-0.94)	0.023
**Severe AAC**	Reference	0.96 (0.40-2.34)	0.37 (0.20-0.69)	0.003

OR (95%CI) for the risk of severe AAC or subclinical AAC was estimated by weighted logistics regression analysis, adjusted for age, sex, race/ethnicity, body mass index, alcohol consumption, physical activity, diabetes, hypertension, TC, TG, HDL-C, eGFR, and lowing lipid agents.

For peer review only - http://bmjopen.bmj.com/site/about/guidelines.xhtml

Table S4. Association between secondhand smoking exposure(SHS) and AAC in never smoker

SHS	Subclinical AAC	p value	Severe AAC	p value
self-report	0.96 (0.20-4.71)	0.958	0.61 (0.29-1.30)	0.183
NNAL	0.61 (0.30-1.26)	0.167	0.73 (0.51-1.03)	0.073
Cotinine	0.84 (0.49-1.44)	0.5	0.96 (0.68-1.35)	0.792

SHS exposure, was defined as self-report, urinary NNAL≥0.001ng/mL, or serum cotinine ≥0.015ng/mL. OR (95%CI) for the risk of severe AAC or subclinical AAC was estimated by weighted logistics regression analysis, adjusted for age, sex, race/ethnicity, body mass index, alcohol consumption, physical activity, diabetes, hypertension, TC, TG, HDL-C, eGFR, and lowing lipid agents.

For peer review only - http://bmjopen.bmj.com/site/about/guidelines.xhtml

For peer review only

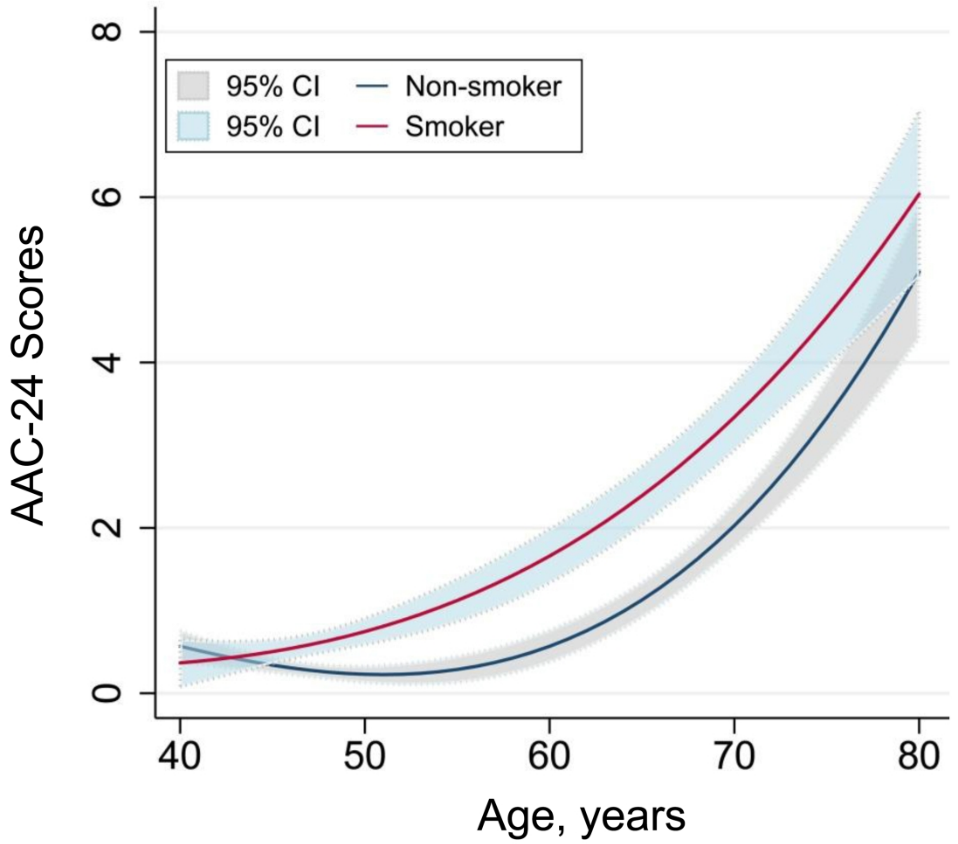

Figure 1. Spline fitting of aged-related AAC in nonsmokers and smokers

112x98mm (1200 x 1200 DPI)

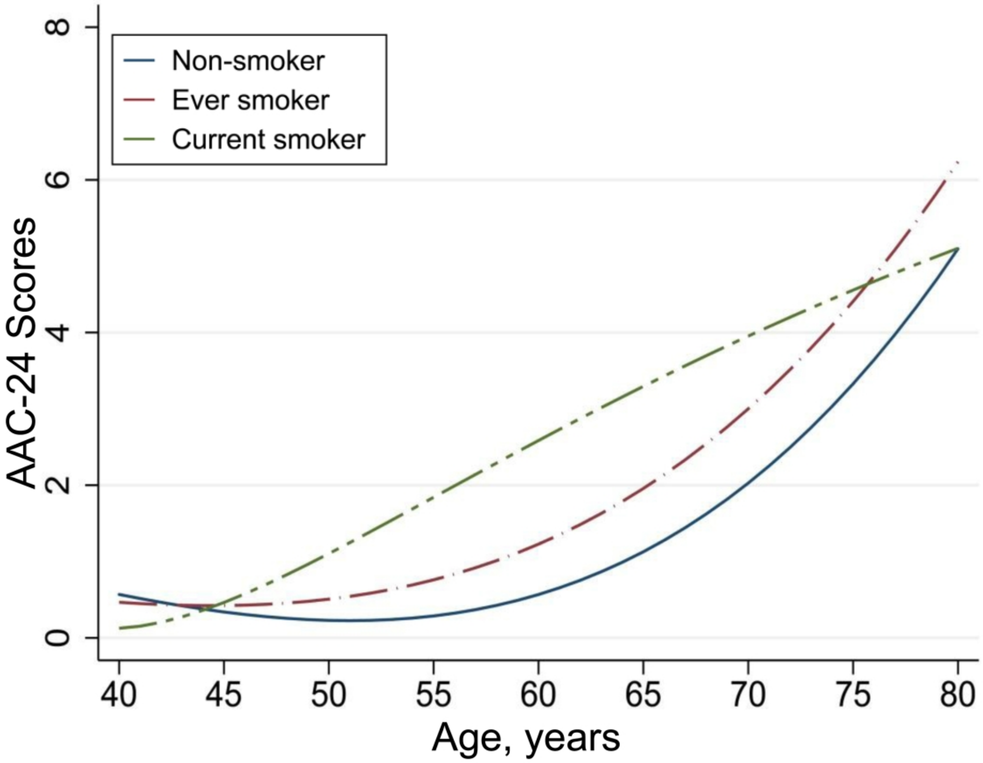

Figure 2. The visualization of age-related AAC in smokers with quit smoking or not

124x96mm (1200 x 1200 DPI)

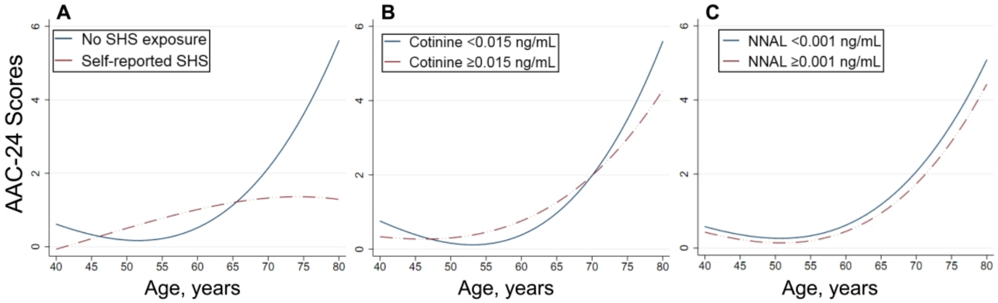

Figure 3. Age-related vascular calcification in nonsmokers with or without secondhand smoking exposure

184x56mm (1200 x 1200 DPI)

Supplemental data

Table S1. Association between age and AAC scores in overall or subgroups by smoking

Models	Overall		Never smoker		Smoker		p for interaction
**AAC-24**	β coefficient (95%CI)	p value	β coefficient (95%CI)	p value	β coefficient (95%CI)	p value	
Crude	0.57 (0.49-0.66)	<0.001	0.49 (0.42-0.57)	<0.001	0.66 (0.53-0.80)	<0.001	0.018
Model 1	0.57 (0.49-0.66)	<0.001	0.50 (0.42-0.58)	<0.001	0.66 (0.52-0.79)	<0.001	0.018
Model 2	0.43 (0.33-0.52)	<0.001	0.37 (0.29-0.44)	<0.001	0.53 (0.38-0.68)	<0.001	0.005
**AAC-8**							
Crude	0.20 (0.17-0.23)	<0.001	0.18 (0.15-0.21)	<0.001	0.22 (0.18-0.26)	<0.001	0.081
Model 1	0.20 (0.17-0.23)	<0.001	0.19 (0.16-0.22)	<0.001	0.22 (0.18-0.26)	<0.001	0.081
Model 2	0.16 (0.12-0.19)	<0.001	0.14 (0.11-0.18)	<0.001	0.18 (0.14-0.23)	<0.001	0.017

β coefficient (95%CI) for AAC scores with a 5-year increase of age. p for interaction was estimated by the survey-weighted Wald test was used to assess the potential interaction between age and smoking for AAC. Model 1 (n=3,140): adjusted for sex, and race/ethnicity. Model 2 (n=2,992): additionally adjusted for body mass index, smoking status, alcohol consumption, physical activity, diabetes, hypertension, TC, TG, HDL-C, eGFR, and lowing lipid agents.

For peer review only - http://bmjopen.bmj.com/site/about/guidelines.xhtml

Table S2. Association between active smoking status and AAC scores

	Never smoking	Quit smoking	Current smoking	
**AAC-8**		**β (95%CI)**	**β (95%CI)**	**p for trend**
Crude	Reference	0.31 (0.17-0.45)	0.23 (0.04-0.43)	0.002
Model 1	Reference	0.15 (0.01-0.29)	0.40 (0.22-0.58)	<0.001
Model 2	Reference	0.14 (0.00-0.29)	0.29 (0.12-0.46)	0.003
**AAC-24**		**β (95%CI)**	**β (95%CI)**	
Crude	Reference	0.92 (0.55-1.29)	0.51 (0.08-0.94)	0.001
Model 1	Reference	0.46 (0.12-0.79)	0.96 (0.54-1.38)	<0.001
Model 2	Reference	0.46 (0.10-0.81)	0.70 (0.34-1.05)	0.001
**Severe AAC**		**OR (95%CI)**	**OR (95%CI)**	
Crude	Reference	2.02 (1.51-2.71)	1.59 (0.94-2.68)	0.012
Model 1	Reference	1.66 (1.18-2.35)	4.30 (2.40-7.71)	<0.001
Model 2	Reference	1.69 (1.12-2.56)	3.39 (2.04-5.66)	<0.001
**Subclinical AAC**		**OR (95%CI)**	**OR (95%CI)**	
Crude	Reference	1.85 (1.37-2.49)	1.73 (1.12-2.66)	0.003
Model 1	Reference	1.47 (1.05-2.07)	2.78 (1.82-4.25)	<0.001
Model 2	Reference	1.46 (1.00-2.14)	2.06 (1.34-3.16)	0.004

β coefficient (95%CI) for AAC scores acorss the smoking status with never smoking as reference. Model 1 (n=3,140): adjusted for age, sex, and race/ethnicity. Model 2 (n=2,992): additionally adjusted for body mass index, alcohol consumption, physical activity, diabetes, hypertension, TC, TG, HDL-C, eGFR, and lowing lipid agents.

For peer review only - http://bmjopen.bmj.com/site/about/guidelines.xhtml

Table S3. Association between smoking cessation and AAC scores in smokers

	Current smoking	Smoking Cessation <10y	Smoking Cessation ≥10y	p for trend
**AAC-24**				
**Subclinical AAC**	Reference	0.97 (0.63-1.48)	0.65 (0.44-0.94)	0.023
**Severe AAC**	Reference	0.96 (0.40-2.34)	0.37 (0.20-0.69)	0.003

OR (95%CI) for the risk of severe AAC or subclinical AAC was estimated by weighted logistics regression analysis, adjusted for age, sex, race/ethnicity, body mass index, alcohol consumption, physical activity, diabetes, hypertension, TC, TG, HDL-C, eGFR, and lowing lipid agents.

For peer review only - http://bmjopen.bmj.com/site/about/guidelines.xhtml

Table S4. Association between secondhand smoking exposure(SHS) and AAC in never smoker

SHS	Subclinical AAC	p value	Severe AAC	p value
self-report	0.96 (0.20-4.71)	0.958	0.61 (0.29-1.30)	0.183
NNAL	0.61 (0.30-1.26)	0.167	0.73 (0.51-1.03)	0.073
Cotinine	0.84 (0.49-1.44)	0.5	0.96 (0.68-1.35)	0.792

SHS exposure, was defined as self-report, urinary NNAL≥0.001ng/mL, or serum cotinine ≥0.015ng/mL. OR (95%CI) for the risk of severe AAC or subclinical AAC was estimated by weighted logistics regression analysis, adjusted for age, sex, race/ethnicity, body mass index, alcohol consumption, physical activity, diabetes, hypertension, TC, TG, HDL-C, eGFR, and lowing lipid agents.

For peer review only - http://bmjopen.bmj.com/site/about/guidelines.xhtml

STROBE Statement—Checklist of items that should be included in reports of ***cross-sectional studies***

	Item No	Recommendation	
**Title and abstract**	1	(*a*) Indicate the study's design with a commonly used term in the title or the abstract	P1
		(*b*) Provide in the abstract an informative and balanced summary of what was done and what was found	P2
**Introduction**			
Background/rationale	2	Explain the scientific background and rationale for the investigation being reported	P5
Objectives	3	State specific objectives, including any prespecified hypotheses	P6
**Methods**			
Study design	4	Present key elements of study design early in the paper	P7
Setting	5	Describe the setting, locations, and relevant dates, including periods of recruitment, exposure, follow-up, and data collection	P7-9
Participants	6	(*a*) Give the eligibility criteria, and the sources and methods of selection of participants	P7
Variables	7	Clearly define all outcomes, exposures, predictors, potential confounders, and effect modifiers. Give diagnostic criteria, if applicable	P8-9
Data sources/ measurement	8*	For each variable of interest, give sources of data and details of methods of assessment (measurement). Describe comparability of assessment methods if there is more than one group	P7
Bias	9	Describe any efforts to address potential sources of bias	P10
Study size	10	Explain how the study size was arrived at	P7
Quantitative variables	11	Explain how quantitative variables were handled in the analyses. If applicable, describe which groupings were chosen and why	P9
Statistical methods	12	(*a*) Describe all statistical methods, including those used to control for confounding	P9-10
		(*b*) Describe any methods used to examine subgroups and interactions	P9-10
		(*c*) Explain how missing data were addressed	
		(*d*) If applicable, describe analytical methods taking account of sampling strategy	
		(*e*) Describe any sensitivity analyses	P9-10
**Results**			
Participants	13*	(a) Report numbers of individuals at each stage of study—eg numbers potentially eligible, examined for eligibility, confirmed eligible, included in the study, completing follow-up, and analysed	P11
		(b) Give reasons for non-participation at each stage	
		(c) Consider use of a flow diagram	
Descriptive data	14*	(a) Give characteristics of study participants (eg demographic, clinical, social) and information on exposures and potential confounders	P11
		(b) Indicate number of participants with missing data for each variable of interest	
Outcome data	15*	Report numbers of outcome events or summary measures	P11-12
Main results	16	(*a*) Give unadjusted estimates and, if applicable, confounder-adjusted estimates and their precision (eg, 95% confidence interval). Make clear which confounders were adjusted for and why they were included	

For peer review only - http://bmjopen.bmj.com/site/about/guidelines.xhtml

		(*b*) Report category boundaries when continuous variables were categorized	P11-12
		(*c*) If relevant, consider translating estimates of relative risk into absolute risk for a meaningful time period	P11-12
Other analyses	17	Report other analyses done—eg analyses of subgroups and interactions, and sensitivity analyses	
**Discussion**			
Key results	18	Summarise key results with reference to study objectives	P13
Limitations	19	Discuss limitations of the study, taking into account sources of potential bias or imprecision. Discuss both direction and magnitude of any potential bias	P16
Interpretation	20	Give a cautious overall interpretation of results considering objectives, limitations, multiplicity of analyses, results from similar studies, and other relevant evidence	P13-15
Generalisability	21	Discuss the generalisability (external validity) of the study results	P16
**Other information**			
Funding	22	Give the source of funding and the role of the funders for the present study and, if applicable, for the original study on which the present article is based	

*Give information separately for exposed and unexposed groups.

Note: An Explanation and Elaboration article discusses each checklist item and gives methodological background and published examples of transparent reporting. The STROBE checklist is best used in conjunction with this article (freely available on the Web sites of PLoS Medicine at http://www.plosmedicine.org/, Annals of Internal Medicine at http://www.annals.org/, and Epidemiology at http://www.epidem.com/). Information on the STROBE Initiative is available at www.strobe-statement.org.

For peer review only

BMJ Open

Modified effect of active or passive smoking on the association between age and abdominal aortic calcification: a nationally representative cross-sectional study

Journal:	*BMJ Open*
Manuscript ID	bmjopen-2020-047645.R2
Article Type:	Original research
Date Submitted by the Author:	22-Aug-2021
Complete List of Authors:	Lv, Luyan; Qujing First People's Hospital, Department of Geriatrics, Qujing First People's Hospital<br>Wu, Shixian; Qujing First People's Hospital<br>Yang, Yungui; Qujing First People's Hospital, Department of Geriatrics<br>yue, xiongli; Qujing First People's Hospital, Department of Geriatrics, Qujing First People's Hospital
**Primary Subject Heading**:	Public health
Secondary Subject Heading:	Cardiovascular medicine, Geriatric medicine, Global health, Smoking and tobacco
Keywords:	Cardiology < INTERNAL MEDICINE, Cardiovascular imaging < RADIOLOGY & IMAGING, Vascular medicine < INTERNAL MEDICINE

SCHOLARONE™
Manuscripts

I, the Submitting Author has the right to grant and does grant on behalf of all authors of the Work (as defined in the below author licence), an exclusive licence and/or a non-exclusive licence for contributions from authors who are: i) UK Crown employees; ii) where BMJ has agreed a CC-BY licence shall apply, and/or iii) in accordance with the terms applicable for US Federal Government officers or employees acting as part of their official duties; on a worldwide, perpetual, irrevocable, royalty-free basis to BMJ Publishing Group Ltd ("BMJ") its licensees and where the relevant Journal is co-owned by BMJ to the co-owners of the Journal, to publish the Work in this journal and any other BMJ products and to exploit all rights, as set out in our licence.

The Submitting Author accepts and understands that any supply made under these terms is made by BMJ to the Submitting Author unless you are acting as an employee on behalf of your employer or a postgraduate student of an affiliated institution which is paying any applicable article publishing charge ("APC") for Open Access articles. Where the Submitting Author wishes to make the Work available on an Open Access basis (and intends to pay the relevant APC), the terms of reuse of such Open Access shall be governed by a Creative Commons licence – details of these licences and which Creative Commons licence will apply to this Work are set out in our licence referred to above.

Other than as permitted in any relevant BMJ Author's Self Archiving Policies, I confirm this Work has not been accepted for publication elsewhere, is not being considered for publication elsewhere and does not duplicate material already published. I confirm all authors consent to publication of this Work and authorise the granting of this licence.

For peer review only

Modified effect of active or passive smoking on the association between age and abdominal aortic calcification: a nationally representative cross-sectional study

Brief Title: Vascular calcification, age, and smoking

Luyan Lv[1†], Shixian Wu[2†], Yungui Yang[1]*, Xiongli Yue[1]*

[1]Department of Geriatrics, Qujing First People's Hospital, Qujing, China;

[2]Ministry of Science and education, Qujing First People's Hospital, Qujing, China;

[†] contributed equally to this article.

Address for correspondence: Xiongli Yue, Yuanlin Road, Qilin District, Qujing, China, 655000, Tel: +86 087 4331 9938, Email: yxl13529891626@163.com; Yungui Yang, Yuanlin Road, Qilin District, Qujing, China, Tel: +86 087 4331 9938, Email: 1369687326@qq.com.

Table number: 2

Figure number: 3

Word count : 2988 (from introduction to conclusion)

For peer review only

For peer review only - http://bmjopen.bmj.com/site/about/guidelines.xhtml

Abstract:

Objectives: The deleterious effects of smoking on atherosclerosis were well-known; however, the interaction among ageing, smoking, and atherosclerosis remains unclear. This study tested the hypothesis that the association between age and vascular calcification, a critical mark of atherosclerosis, was modified by smoking.

Design: Cross-sectional study

Setting: A nationally representative sample, NHANES 2013–2014.

Participants: This study included 3140 adults aged 40-80 years with eligible data for abdominal aortic calcification (AAC). Active and passive smoking exposure was identified through self-reports and tobacco metabolites (serum cotinine and urinary 4-methylnitrosamino-3-pyridyl-1-butanol).

Primary outcome measures: AAC score was determined using dual-energy X-ray absorptiometry (DXA) scans. Odds ratio (OR) was estimated using the logistic regression method to assess the association between age and the presence of severe or subclinical AAC stratified by smoking exposure. The survey-weighted Wald test was used to evaluate potential interactions.

Results: AAC was positively associated with age in the general population. After adjustment for age, sex, race/ethnicity, and other cardiovascular risk factors, age was significantly associated with the odds of severe AAC (OR for each 5-year increase in age: 1.66, 95% CI: 1.48-1.87, $p<0.001$). As expected, the association between age and vascular calcification was especially stronger in smokers than in never smokers (p for interaction ≤ 0.014). According to spline fitting, the progression of vascular

For peer review only

calcification was significantly increased after 45 years in smokers compared with that after 60 years in never smokers. Quitting smoking may compromise the deleteriousness of the vascellum especially in younger adults. However, the difference in age-related calcification among never smokers with or without secondhand smoke exposure was minor, regardless of the definition by self-report, serum cotinine, or urinary 4-(methylnitrosamino)-1-(3-pyridyl)-1-butanol.

Conclusions: Smoking significantly accelerated the progression of age-related subclinical atherosclerosis. Early smoking cessation should be encouraged among young smokers. The effect of passive smoking exposure on arteriosclerosis should be assessed further.

Keywords:

Abdominal aortic calcification, arteriosclerosis, age, smoking, second-hand smoking

For peer review only - http://bmjopen.bmj.com/site/about/guidelines.xhtml

Strengths and limitations of this study

1. This study involves a nationally representative sample of the adult population of the United States, suggesting an advantageous generalisation of the findings.
2. Data were collected using standardised and validated protocols, which suggested favourable repeatability.
3. This study evaluated the potential modification for age-related abdominal aortic calcification by active smoking or secondhand smoke exposure, defined by self-reports or serum nicotine metabolites.
4. This study was based on a cross-sectional design, which was less likely to conclude the causality.
5. Abdominal aortic calcification measurements were only performed in participants aged 40 years or more. Whether smoking influences age-related vascular calcification in adults aged < 40 years warrants further investigation.

For peer review only - http://bmjopen.bmj.com/site/about/guidelines.xhtml

Introduction

Cardiovascular disease (CVD) has been the leading cause of death worldwide since the early 20th century, imposing a heavy burden on public health and healthcare systems.[1] Atherosclerosis has always been the primary pathological basis of CVD among the wide array of underlying cardiovascular lesions.[2] Early identification of populations at high risk for subclinical atherosclerosis is important to prevent the progression of CVD.[3] Abdominal aortic calcification (AAC) is considered a key hallmark of atherosclerosis and an independent predictor of subsequent cardiovascular morbidity and mortality.[1, 4] Evidence from previous studies suggests that severe AAC is associated with a higher risk of major cardiovascular events, thus reflecting a poor cardiovascular health status.[5]

Epidemiological studies have demonstrated that vascular calcification or atherosclerosis is a common physiological process associated with ageing.[6, 7] With the improvement in the primary and secondary prevention of cardiovascular events, the association between age and aortic calcification during the contemporary era remains unknown. Smoking is a well-established modifiable risk factor for atherosclerosis and CVD via several underlying pathways,[8] and it has been a critical environmental hazard for public health.[9] It is noteworthy that worldwide, approximately 11% of deaths related to CVD are attributed to smoking exposure.[10] Furthermore, secondhand smoke (SHS) exposure in non-smokers has also been associated with CVD and other health consequences.[11] Although several studies had demonstrated the associations between ageing or smoking and atherosclerotic events, the evidence on the interactive

For peer review only - http://bmjopen.bmj.com/site/about/guidelines.xhtml

effect between ageing and active or passive smoking in aortic calcification is limited.[12] Clarifying this issue may provide new insights into the atherosclerotic process and identify the targeted population who could potentially benefit from smoking cessation education. In this context, this study tested the hypothesis that the association between age and AAC was modified by smoking exposure, including active and secondhand smoking, in a nationally representative sample of the middle-aged and elderly population.

For peer review only - http://bmjopen.bmj.com/site/about/guidelines.xhtml

Methods

Study Population

The data were extracted from the National Health and Nutrition Examination Surveys (NHANES) 2013–2014, an ongoing nationally representative-, stratified-, multistage probability-sampling survey designed by the Center for Disease Control and Prevention of the United States. The methods and protocols have been described in previous studies.[13, 14] There were 10,175 individuals in NHANES 2013–2014, and 3815 of them been adults aged 40 years of age and above. Pregnant women were excluded from the study (n=3). Dual-energy X-ray absorptiometry (DXA) scans were conducted for participants aged 40 years and above. Aortic calcification adjacent to the lumbar vertebrae L1–L4 was assessed. Participants without a scan (n=482) or with image invalidity (n=190) were excluded. All adults with eligible AAC scores were included in this study (n = 3140).[15] This study was conducted in accordance with the Declaration of Helsinki. The protocols of the NHANES were approved by the Ethics Review Board of the National Center for Health Statistics. All participants provided informed consent. . Detailed information is available on the website http://www.cdc.gov/nchs/nhanes/irba98.htm.

AAC measurement

AAC could be easily assessed on lateral spine DXA scans.[16] The scores were calculated from a lateral scan of the lumbar spine (L1-L4 vertebrae) that was acquired using the Hologic Discovery model A densitometers (Hologic, Inc., Marlborough,

For peer review only - http://bmjopen.bmj.com/site/about/guidelines.xhtml

Massachusetts), and Apex software (version3.2).[1, 16] The radiation exposure from DXA for the lateral spine scan was extremely low ((<20 uSv). All scans were viewed using the Optasia SpinAnalyzer software. The 24-point semi-quantitative score (AAC-24, Kauppila score) was used for the primary analysis, and the AAC-8 score (Schousboe score) was used for sensitivity analysis.[16] Both methods have been validated with favourable sensitivity and specificity to reflect the severity of AAC. AAC-24 was calculated according to the length of calcification at the posterior and anterior aortic walls contiguous to the L1-L4 lumbar vertebrae. Treating lines across the middle of the intervertebral spaces as segment boundaries, the abdominal aorta was divided into eight segments. AAC was scored from 0 to 3 according to calcification length in the aortic wall of each segment (0 point: no calcification; 1 point: ≤1/3 arterial wall in each segment; 2 points: 1/3– 2/3; 3 points: >2/3). AAC-8 is a simplified method derived from the AAC-24. AAC-8 in the anterior or posterior aortic walls in front of the L1-L4 was scored 0 to 4(0 point: no calcification; 1 point: no more than the length of one vertebra; 2 points: no more than the length of 2 vertebrae; 3 points: no more than the length of 3 vertebrae; and 4 points: more than the length of three vertebrae). Therefore, small calcifications influence the AAC-8 score =less by but need more skilled technologists compared with AAC-24. DXA examinations were performed by trained and certified radiology technologists. Detailed protocols have been presented in previous studies.[1, 16]

For peer review only - http://bmjopen.bmj.com/site/about/guidelines.xhtml

Smoking exposure and cigarette biomarker assessment

Smoking was defined as smoking at least 100 cigarettes during his or her life, ascertained through self-reports including both past and current cigarette smoking status. Tobacco metabolites, serum cotinine, and urinary 4-(methylnitrosamino)-1-(3-pyridyl)-1-butanol (NNAL) were considered as biomarkers of passive smoking exposures in nonsmokers.[17] Three definitions were used to identify individuals with SHS exposure, including self-report, urinary NNAL ≥0.001ng/mL, or serum cotinine level ≥0.015ng/mL, as previously mentioned.[18] Serum cotinine and urinary NNAL were determined by high-performance liquid chromatography-tandem mass spectrometric (HPLC-MS), with atmospheric pressure chemical ionisation or electrospray ionisation, respectively. The biosample was pre-treated with methyl-D3-cotinine or 4-(methylnitrosoamino)-1-(3-pyridyl)-1-butanol-1,2',3',4',5',6'-13C[6] as the internal standard. A C18 HPLC column was used to separate metabolites. The eluents from these injections were monitored using a mass spectrometer. The m/z 80 product ion from the m/z 177 quasi-molecular ion was used to quantify serum cotinine.

Demographic, clinical, and laboratory variables

Data concerning age, sex, race/ethnicity, smoking status, physical activity, and alcohol consumption, and history of chronic diseases were collected using standardized questionnaires during personal interviews.[13] Physical examinations were conducted at the Mobile Examination Centre (MEC) with standardised protocols. Peripheral blood was collected from the MEC and analysed in the central laboratory,

For peer review only - http://bmjopen.bmj.com/site/about/guidelines.xhtml

according to standardised procedures. Race/ethnicity was categorised into four groups: non-Hispanic white, non-Hispanic black, Hispanic-Mexican, and others. Physical activity was categorised as vigorous, moderate, and less active as presented elsewhere.[19] Drinking was assessed via questionnaires for the preceding year. One drink was defined as 12 -oz beer, 4 -oz of wine, or 1 oz of liquor, each covering approximately 10 g of alcohol.[19] Body mass index (BMI) was calculated as weight divided by height squared (kg/m2). Subjects' triglyceride (TG), total cholesterol (TC), high-density lipoprotein-cholesterol (HDL-C), and serum creatinine levels were determined in morning fasting status. The estimated glomerular filtration rate (eGFR) was calculated using the Chronic Kidney Disease Epidemiology Collaboration.[13] Hypertension was defined as a self-reported diagnosis by a doctor, systolic blood pressure ≥140 mmHg, or diastolic blood pressure ≥90 mmHg. Diabetes was defined as receiving anti-hyperglycemic therapy or HbA1c levels of ≥6.5%.[19]

Statistical Analysis

Continuous and categorical variables are presented as means and percentages, weighted with primary sampling units, pseudo-strata, and appropriate sampling weights to account for the complex sampling design. Crude and multivariable logistic or linear regression models were applied to assess the association between age and AAC scores. According to previous studies,[1, 16] severe AAC was defined as AAC-24 score of ≥6 points and subclinical AAC was defined as AAC-24 score of ≥2 points. Logistic regression analysis was used to calculate odds ratios as primary analysis. Two adjusted models were used in this study. Model 1 was adjusted for sex, and

For peer review only - http://bmjopen.bmj.com/site/about/guidelines.xhtml

race/ethnicity. Model 2 was additionally adjusted for BMI, smoking status, alcohol consumption, physical activity, diabetes, hypertension, TC, HDL-C, estimated glomerular filtration rate, and decreasing lipid agents. The variance inflation factor of each variable was not more than 2.64, which did not suggest a multicollinearity of covariates. The survey-weighted Wald test was used to assess the potential interaction between age and smoking for AAC. The β coefficient (95% confidence interval [CI]) was presented as the increased scores of AAC-24 or AAC-8 for each 5-year increase. Two-way fractional polynomial regression spline plots were used to visualise the relationship between age and vascular calcification among smokers or never smokers, and SHS or non-SHS. Statistical analysis was conducted with Stata (version SE15.0). All tests with a 2-sided p value of less than 0.05 were considered statistically significant.

Patient and public involvement

No patients were involved in the development of the research question or design of the study.

For peer review only - http://bmjopen.bmj.com/site/about/guidelines.xhtml

Results

Overall, there were 3140 adults aged ≥40 years with AAC scores in NHANES 2013–2014 (**Table 1**). The weighted mean age was 57.4 years and 51.9% (n= 1622) were women. According to self-report, 54.0% were never smokers and 46.0% were smokers, including 28.2% ever smokers and 17.8% current smokers. Compared with participants aged ≤65 years, older participants had higher proportions of non-Hispanic whites, quitting smoking, diabetes, and cardiovascular medications, as well as less physical activity and lower renal function.

The association between age and AAC in all the participants, stratified by smoking, was shown in **Table 2 and Supplemental Table 1**. In univariable and sex- and race-adjusted logistic regression models, age was significantly associated with severe or subclinical ACC, with 85% and 44% increased odds for each 5-year increase in age (each p<0.001). After adjustment for sex, race, smoking, physical activity, alcohol intakes, BMI, hypertension, diabetes, TC, TG, HDL-C, eGFR, and decreasing-lipid agents, the association of age and AAC remained significant with ORs (95% CI) of 1.66 (1.48–1.87) and 1.37 (1.25–1.50) for each 5-year increase in age (each p<0.001), respectively (**Table 2)**. In addition, AAC-24 and AAC-8 scores were positively associated with age **(Supplemental Table 1)**. The association between age and AAC-24 score remained significant (β coefficient for each 5-year increase in age: 0.43, 95% CI: 0.33-0.52, p<0.001). Supplementary analysis for the association between smoking exposure and AAC was conducted (**Supplemental Table 2-4**). As expected, current smoking was significantly associated with AAC severity compared

For peer review only - http://bmjopen.bmj.com/site/about/guidelines.xhtml

to never smokers. Treating current smokers as a reference, participants with smoking cessation more than 10 years had lower AAC scores. However, the relationship between passive smoking and AAC was not observed.

Because smoking cigarettes are a strong risk factor for vascular injury, we assessed age-related aortic calcification in subgroups according to smoking status (**Table 2 and Supplemental Table 1**). As expected, the effect modification by smoking was observed (p for interaction ≤0.014). In fully adjusted models, the OR (95%CIs) for each 5-year increase in age for severe AAC was 1.67 (1.50–1.85) among non-smoking participants versus 2.03 (1.70–2.42) among smoking participants. This suggests that the ever and current smoking may accelerate age-related AAC by 30% compared with never smoking status. Repeated analysis was performed using binary subclinical AAC, and continuous AAC-24 and AAC-8 scores, and the correlations remained unchanged. According to spline fittings (**Figure 1**), never smokers had a flat trend of age-related calcification before 65 years, whereas the calcification was significantly advanced after 45 years in smoking participants. Furthermore, compared with current smokers, participants who quit smoking had lower scores for age-related calcification, especially for those aged 50-70 years (**Figure 2**). However, among never smokers, SHS exposure did not significantly affect age-related AAC (**Figure 3**).

For peer review only

Discussion

In this nationally representative sample from the general population of the United States in 2013–2014, the association between AAC and age differed depending on the smoking status after adjustment for various confounders. Age-related calcification was flat before 60 years old, whereas it was driven by smoking after 45 years old. Compared with persistent smoking, quitting smoking may mitigate the process of age-induced calcification, especially for adults aged 45-70 years. The deleterious effect of secondhand smoking on vascular calcification in adults was not observed in this study. Our study is of particular interest because our findings highlight the benefits of never smoking and early quit smoking in the process of age-related vascular calcification or atherosclerosis in the contemporary population.

Vascular calcification was considered to be a degenerative and end-stage consequence of the ageing process.[6] Arterial calcification was detected in approximately one-fourth of the populations aged 50 years. This proportion subsequently increased to beyond 60% in the population aged more than 75 years.[7] Our results also consistently noted that AAC scores slowly increased in middle-aged participants, and robustly increased in the elderly participants aged $\geq$ 65 years.

Vascular calcification has been linked with vascular injury and subclinical atherosclerotic disease.[16, 20] Vascular wall calcification is a common byproduct of atherosclerotic plaques and was thus considered as a potential marker of subclinical atherosclerosis as well as a simple tool for cardiovascular risk stratification.[16] Although previous reports have highlighted the implications of AAC as a biomarker

For peer review only - http://bmjopen.bmj.com/site/about/guidelines.xhtml

of cardiovascular morbidity and mortality in adults,[21-23] AAC has received less attention as a modifiable marker in preventing atherosclerosis and cardiovascular risk.[24] Our findings suggest that quitting smoking is a simple and effective means to mitigate the process of arterial calcification and atherosclerosis in the general population, especially in young adults. Lifestyle changes seem to be promising, low-cost, and easy to spread.

Hisamatsu et al. conducted an interesting study and demonstrated a positive association between smoking and subclinical atherosclerosis that was comprehensively assessed based on coronary artery calcification, carotid intima-media thickness and plaque, aortic artery calcification (from the aortic arch to iliac bifurcation), and ankle-brachial index.[25] The relationship attenuated over the time of smoking cessation Moreover, Pham et al. supplemented the analysis for the relationship between smoking habits and the progression of coronary and aortic artery calcification in a follow-up study.[26] These findings supported that smoking is a strong risk factor for coronary calcification and atherosclerosis. Jung and colleagues observed the positive association between AAC and long-term smoking exposure in 218 men aged 40–81 years. Even a low dosage of smoking increased the risk of AAC.[27] Furthermore, Hirooka and colleagues analysed 313 Japanese and 302 Korean men aged 40–49 years and concluded that smoking with higher pack-years was significantly associated with artery calcium, suggesting a dose-response pattern between cigarette use and progression of atherosclerosis.[28] A small sample size and selection bias may limit the extrapolation. Our analysis also supported the increased

For peer review only - http://bmjopen.bmj.com/site/about/guidelines.xhtml

risk of severe or subclinical angiosteosis associated with smoking. Notably, smoking cessation for more than 10 years could partly attenuate the harm of smoke exposure, which was consistent with the findings of previous reports.[25, 26, 29] Our results further emphasised that the association between age and ACC was modified by smoking and that the detrimental effects of ageing and smoking were not simply superposed.

Based on NHANES data, a recent study noted that serum cotinine levels, a biomarker of smoking, were associated with the risk of severe AAC in 2840 adults.[16] However, circulating cotinine only reflects short-term exposure to smoking, and the interaction between smoking and age on the process of vascular calcification remains unknown. Our results highlighted that the process of age-related calcification was slow before 60 years of age. In contrast, smoking, especially persistent smoking, significantly accelerated this process since 40 years of age. Quitting smoking at an early stage may at least partially moderate the speed for adults aged ≤70 years. To the best of our knowledge, our study is the first study that specifically investigates the interaction between age and smoking on the severity of aortic calcification in humans. Notably, previous studies have been established the association between smoking and vascular calcification, suggesting the potential benefits of quit smoking to prevent atherosclerosis.[8, 16, 30, 31] However, the complex interaction with the aging process needs to be clarified, which may provide new insights into the optimal time to prevent atherosclerosis by tobacco control.[30] Our results suggest small benefits of quit smoking in the elderly aged >70 years, which highlights the importance of early action to reduce the burden of vascular calcification.

For peer review only - http://bmjopen.bmj.com/site/about/guidelines.xhtml

SHS exposure has been proven to be associated with CVD and adverse outcomes.[11] Approximately 85% of SHS was derived from side-stream smoke that seemed potentially more harmful due to non-filtering.[12] As another purpose, we investigated the relationship between AAC and SHS based on self-reports, and serum cotinine or urine NNAL in never smokers. However, the neutral link between arterial calcification and passive smoking that we noted might be not the ultimate conclusion. The questionnaires of secondhand smoking were only designed for the preceding 7 days, although we used the definition of potentially persistent SHS exposure in indoor settings at job or home. Serum cotinine and urinary NNAL, as more accurate biomarkers of smoking exposure than self-reports, have been validated to estimate the SHS exposure in the past 2–4 days and 6–12 weeks, respectively.[18] The relationship was also insignificant in stratifications by serum cotinine or urinary NNAL. Both biomarkers only reflected exposures over the past several days or weeks, which were insufficient to reflect long-term exposure. The progression of vascular calcification is a chronic process, even beyond the years. Therefore, further studies may need multiple repeated measurements or questionnaires over the years to assess the effect of SHS on age-related calcification.

Limitations and strengths

Our study has some strengths that support favourable repeatability, including a nationally representative sample of the adult population, standardised and validated protocols, and full adjustment for a large number of potential confounders. However, our conclusions should be explained in consideration of the following limitations.

For peer review only - http://bmjopen.bmj.com/site/about/guidelines.xhtml

First, this study was based on a cross-sectional design, which was less likely to conclude the causality. More convincing evidence could be obtained from a longitudinal study with multiple measurements. Second, the analysis of the relationship between secondhand smoke and AAC was not the ultimate seal. The duration of SHS exposure should be considered in future studies. Third, AAC measurement was performed only in the population aged 40 years or above. Whether smoking influences age-related vascular calcification in adults aged <40 years old that warrants investigation. Fourth, residual confounding factors could not be eliminated, although we strictly adjusted for 16 covariates in the regression model.

Conclusions

Our results indicate that smoking significantly modifies the association between age and AAC severity in adults aged 40 years or above. Age-related vascular calcification is accelerated by smoking, which is partly reduced by smoking cessation. However, whether secondhand smoke affects this process requires further investigation.

For peer review only - http://bmjopen.bmj.com/site/about/guidelines.xhtml

Acknowledgments: The authors would like to thank all participants and staff in NHANES for their great contributions.

Data availability statement: All data came from the NHANES study (http://www.cdc.gov/nchs/nhanes). Data are available on reasonable request from the CDC of of United States or corresponding authors.

Ethics Approval: The protocols and procedures of the NHANES study were agreed by the Research Ethics Review Board of the Centers of Disease Control and Prevention of United States (Protocol Number. 2011-17).

Conflict of interest: None declared.

Patient consent for publication: Not required.

Sources of Funding: This manuscript was written independently. The authors received no financial support.

Author contributions:

Yungui Yang contributed to the conceptualisation. Yungui Yang and Xiongli Yue developed the protocols. Luyan Lv and Shixian Wu organized and analyzed all data. Xiongli Yue was responsible for the project administration. All authors wrote and reviewed the manuscript. Yungui Yang and Xiongli Yue take the responsibility for the integrity and accuracy of data.

For peer review only - http://bmjopen.bmj.com/site/about/guidelines.xhtml

References:

1 Li YW, Chen WL. Clinical relevance of serum uric acid and abdominal aortic-calcification in a national survey. Clin Cardiol. 2020.

2 Lusis AJ. Atherosclerosis. Nature. 2000;**407**(6801):233-241.

3 Kalsch H, Lehmann N, Moebus S, et al. Aortic Calcification Onset and Progression: Association With the Development of Coronary Atherosclerosis. J Am Heart Assoc. 2017;**6**(4).

4 Wilson PW, Kauppila LI, O'Donnell CJ, et al. Abdominal aortic calcific deposits are an important predictor of vascular morbidity and mortality. Circulation. 2001;**103**(11):1529-1534.

5 Szulc P. Abdominal aortic calcification: A reappraisal of epidemiological and pathophysiological data. Bone. 2016;**84**:25-37.

6 Zhang B, Miller VM, Miller JD. Influences of Sex and Estrogen in Arterial and Valvular Calcification. Front Endocrinol (Lausanne). 2019;**10**:622.

7 Cannata-Andia JB, Rodriguez-Garcia M, Carrillo-Lopez N, Naves-Diaz M, Diaz-Lopez B. Vascular calcifications: pathogenesis, management, and impact on clinical outcomes. J Am Soc Nephrol. 2006;**17**(12 Suppl 3):S267-S273.

8 Wang Z, Wang D, Wang Y. Cigarette Smoking and Adipose Tissue: The Emerging Role in Progression of Atherosclerosis. Mediators Inflamm. 2017;**2017**:3102737.

9 Smoking prevalence and attributable disease burden in 195 countries and territories, 1990-2015: a systematic analysis from the Global Burden of Disease

For peer review only - http://bmjopen.bmj.com/site/about/guidelines.xhtml

Study 2015. Lancet. 2017;**389**(10082):1885-1906.

10 Ezzati M, Henley SJ, Thun MJ, Lopez AD. Role of smoking in global and regional cardiovascular mortality. Circulation. 2005;**112**(4):489-497.

11 Asfar T, Koru-Sengul T, Ruano-Herreria EC, Sierra D, Lee DJ, Arheart KL. Secondhand Smoke Exposure Among High-Risk Patients in the United States (NHANES 2001-2012): Implications for Clinical Practice. Nicotine Tob Res. 2019;**21**(4):551-556.

12 Park YS, Lee CH, Kim YI, et al. Association between secondhand smoke exposure and hypertension in never smokers: a cross-sectional survey using data from Korean National Health and Nutritional Examination Survey V, 2010-2012. BMJ Open. 2018;**8**(5):e21217.

13 Wang S, Liu Y, Liu J, et al. Mitochondria-derived methylmalonic acid, a surrogate biomarker of mitochondrial dysfunction and oxidative stress, predicts all-cause and cardiovascular mortality in the general population. Redox Biol. 2020;**37**:101741.

14 Rossato LT, de Branco F, Azeredo CM, Rinaldi A, de Oliveira EP. Association between omega-3 fatty acids intake and muscle strength in older adults: A study from National Health and Nutrition Examination Survey (NHANES) 1999-2002. Clin Nutr. 2020.

[dataset] 15 Kathryn Porter. Data from: The National Health and Nutrition Examination Survey (NHANES). 2013-2014. The Centers for Disease Control and Prevention of United States, September 30, 2018.

For peer review only

For peer review only - http://bmjopen.bmj.com/site/about/guidelines.xhtml

https://www.cdc.gov/nchs/nhanes/.

16 Wen X, Xia Y, Guo M, Zhao L, Zhou L. Association Between Serum Cotinine and Severe Abdominal Aortic Calcification in US Adults. Angiology. 2020;**71**(4):333-339.

17 Fu Z, Jiang H, Xu Z, Li H, Wu N, Yin P. Objective secondhand smoke exposure in chronic obstructive pulmonary disease patients without active smoking: the U.S. National Health and Nutrition Examination Survey (NHANES) 2007-2012. Ann Transl Med. 2020;**8**(7):445.

18 Fu Z, Jiang H, Xu Z, Li H, Wu N, Yin P. Objective secondhand smoke exposure in chronic obstructive pulmonary disease patients without active smoking: the U.S. National Health and Nutrition Examination Survey (NHANES) 2007-2012. Ann Transl Med. 2020;**8**(7):445.

19 Wang S, Tian W, Liu Y, et al. Temporal trend of circulating trans-fatty acids and risk of long-term mortality in general population. Clin Nutr. 2020.

20 Budoff MJ, Achenbach S, Blumenthal RS, et al. Assessment of coronary artery disease by cardiac computed tomography: a scientific statement from the American Heart Association Committee on Cardiovascular Imaging and Intervention, Council on Cardiovascular Radiology and Intervention, and Committee on Cardiac Imaging, Council on Clinical Cardiology. Circulation. 2006;**114**(16):1761-1791.

21 Bastos GF, Voute MT, Hoeks SE, et al. Calcification of the abdominal aorta as an independent predictor of cardiovascular events: a meta-analysis. Heart.

For peer review only - http://bmjopen.bmj.com/site/about/guidelines.xhtml

2012;**98**(13):988-994.

22 Martino F, Di Loreto P, Giacomini D, et al. Abdominal aortic calcification is an independent predictor of cardiovascular events in peritoneal dialysis patients. Ther Apher Dial. 2013;**17**(4):448-453.

23 Bolland MJ, Wang TK, van Pelt NC, et al. Abdominal aortic calcification on vertebral morphometry images predicts incident myocardial infarction. J Bone Miner Res. 2010;**25**(3):505-512.

24 Golledge J. Abdominal aortic calcification: clinical significance, mechanisms and therapies. Curr Pharm Des. 2014;**20**(37):5834-5838.

25 Hisamatsu T, Miura K, Arima H, et al. Smoking, Smoking Cessation, and Measures of Subclinical Atherosclerosis in Multiple Vascular Beds in Japanese Men. J Am Heart Assoc. 2016;**5**(9).

26 Kianoush S, Yakoob MY, Al-Rifai M, et al. Associations of Cigarette Smoking With Subclinical Inflammation and Atherosclerosis: ELSA-Brasil (The Brazilian Longitudinal Study of Adult Health). J Am Heart Assoc. 2017;**6**(6).

27 Jung JG, Wu LT, Kim JS, Kim ED, Yoon SJ. Relationship between Smoking and Abdominal Aorta Calcification on Computed Tomography. Korean J Fam Med. 2019;**40**(4):248-253.

28 Hirooka N, Kadowaki T, Sekikawa A, et al. Influence of cigarette smoking on coronary artery and aortic calcium among random samples from populations of middle-aged Japanese and Korean men. J Epidemiol Community Health. 2013;**67**(2):119-124.

For peer review only - http://bmjopen.bmj.com/site/about/guidelines.xhtml

29 McEvoy JW, Nasir K, DeFilippis AP, et al. Relationship of cigarette smoking with inflammation and subclinical vascular disease: the Multi-Ethnic Study of Atherosclerosis. Arterioscler Thromb Vasc Biol. 2015;**35**(4):1002-1010.

30 Lehmann N, Mohlenkamp S, Mahabadi AA, et al. Effect of smoking and other traditional risk factors on the onset of coronary artery calcification: results of the Heinz Nixdorf recall study. Atherosclerosis. 2014;**232**(2):339-345.

31 Rasmussen T, Frestad D, Kober L, et al. Development and progression of coronary artery calcification in long-term smokers: adverse effects of continued smoking. J Am Coll Cardiol. 2013;**62**(3):255-257.

For peer review only

Tables and figure legends

Table 1. Characteristics of the Study Population in NHANES 2013-2014.

Variable	Overall	Age ≤65 years	Age >65 years
Age ,year	57.38 (56.75-58.01)	51.89 (51.39-52.39)	73.18 (72.72-73.65)
Male ,%	48.08	49.3	44.56
Race/ethnicity, %			
Hispanic-Mexican	6.98	8.03	3.98
Other Ethnicity	11.89	13.0	8.69
Non-Hispanic White	71.01	68.2	79.1
Non-Hispanic Black	10.12	10.78	8.23
Smoking status, %			
Never smoking	53.98	54.38	52.84
Former smoker	28.15	24.27	39.33
Current smoker	17.87	21.35	7.831
Physical activity, %			
Inactive	48.34	45.91	55.31
Moderate	32.75	31.67	35.86
Vigorous	18.91	22.41	8.83
Alcohol intakes, g	4.69 (4.01-5.36)	5.31 (4.45-6.17)	2.98 (2.39-3.56)
BMI, kg/m^2	28.54 (28.17-28.9)	28.7 (28.25-29.15)	28.07 (27.56-28.58)
Triglycerides, mmol/L	1.81 (1.73-1.89)	1.84 (1.74-1.94)	1.74 (1.63-1.85)
Total cholesterol, mmol/L	5.09 (5.04-5.13)	5.18 (5.12-5.23)	4.83 (4.70-4.96)
HDL-C, mmol/L	1.42 (1.40-1.43)	1.41 (1.38-1.43)	1.44 (1.39-1.48)

For peer review only - http://bmjopen.bmj.com/site/about/guidelines.xhtml

eGFR ,mL/min per 1.73m²	84.28 (83.28-85.28)	89.49 (88.26-90.72)	69.47 (68.52-70.42)
SBP, mmHg	125.42 (124.31-126.53)	122.66 (121.43-123.89)	133.28 (131.63-134.92)
DBP, mmHg	70.75 (69.8-71.71)	72.82 (72.03-73.61)	64.86 (62.81-66.91)
Diabetes, %	14.32	11.39	22.74
Lowering lipid, %	30.02	21.78	53.74
ACEI/ARBs, %	26.87	20.23	45.97
Beta-blockers, %	14.63	8.89	31.14

Statistics of variables are presented as weighted proportion (%) or means (CI%). Abbreviation: ACEI, angiotensin-converting enzyme inhibitor; ARBs, angiotensin II receptor antagonist; BMI, body mass index; SBP, systolic blood pressure; DBP, diastolic blood pressure; eGFR, estimated glomerular filtration rate; HDL-C, high-density lipoprotein cholesterol.

For peer review only - http://bmjopen.bmj.com/site/about/guidelines.xhtml

Table 2. Association between age and severe or subclinical AAC in all participants or subgroups by smoking

Models	**Overall**		**Never smoker**		**Smoker**		p for interaction
**Severe AAC**	**OR (95%CI)**	p value	**OR (95%CI)**	p value	**OR (95%CI)**	p value	
Crude	1.84 (1.68-2.02)	<0.001	1.67 (1.50-1.85)	<0.001	2.03 (1.70-2.42)	<0.001	0.008
Model 1	1.85 (1.69-2.02)	<0.001	1.67 (1.50-1.85)	<0.001	2.07 (1.74-2.46)	<0.001	0.008
Model 2	1.66 (1.48-1.87)	<0.001	1.55 (1.32-1.82)	<0.001	1.78 (1.52-2.09)	<0.001	0.014
**Subclinical AAC**							
Crude	1.44 (1.32-1.56)	<0.001	1.29 (1.17-1.44)	<0.001	1.55 (1.43-1.68)	<0.001	0.002
Model 1	1.44 (1.32-1.57)	<0.001	1.29 (1.16-1.43)	<0.001	1.55 (1.45-1.68)	<0.001	0.001
Model 2	1.37 (1.25-1.50)	<0.001	1.26 (1.14-1.39)	<0.001	1.45 (1.31-1.61)	<0.001	0.011

The 24-point semi-quantitative score of abdominal aortic calcification (AAC-24, Kauppila score) was calculated according to the length of calcification at the posterior and anterior aortic walls contiguous to the L1-L4 lumbar vertebrae. Treating lines across the middle of the intervertebral spaces as segment boundaries, the abdominal aorta was divided into eight segments. AAC was scored from 0 to 3 according to calcification length in the aortic wall of each segment (0 point: no calcification; 1 point: ≤1/3 arterial wall in each segment; 2 points: 1/3– 2/3; 3 points: >2/3). Severe AAC was defined as AAC-24 scores ≥6 points and subclinical AAC was defined as AAC-24 scores ≥2 points. The odds ratio (OR) (95% confidence interval [CI]) for the presence of severe or subclinical AAC was estimated using weighted logistic regression analysis. p value for interaction was used to assess the potential interaction between age and smoking for AAC using the survey-weighted Wald

For peer review only - http://bmjopen.bmj.com/site/about/guidelines.xhtml

test. Model 1: adjusted for sex, and race/ethnicity; Model 2: additionally adjusted for body mass index, smoking status, alcohol consumption, physical activity, diabetes, hypertension, TC, TG, HDL-C, eGFR, and decreasing lipid agents.

For peer review only

For peer review only - http://bmjopen.bmj.com/site/about/guidelines.xhtml

Figure 1. Spline fitting of aged-related AAC in never smokers and smokers

The spline curve shows the relationship between scores of AAC-24 and age in smokers and never smokers based on fractional polynomial regression. The solid lines represent point estimates, and dashed lines represent 95% CIs. AAC-24 was calculated according to the length of calcification at the posterior and anterior aortic walls contiguous to the L1-L4 lumbar vertebrae. Treating lines across the middle of the intervertebral spaces as segment boundaries, the abdominal aorta was divided into eight segments. AAC was scored from 0 to 3 according to calcification length in the aortic wall of each segment (0 point: no calcification; 1 point: ≤1/3 arterial wall in each segment; 2 points: 1/3– 2/3; 3 points: >2/3). Never smokers had a flat trend of age-related calcification before the age of 65, whereas the calcification was significantly advanced after the age of 45 in subjects with smoking.

For peer review only - http://bmjopen.bmj.com/site/about/guidelines.xhtml

Figure 2. Visualization of age-related AAC in smokers who have quit smoking or not

The spline curve shows the relationship between scores of AAC-24 and age in smokers with quit smoking or not based on fractional polynomial regression. The solid lines represent point estimates.

For peer review only

For peer review only - http://bmjopen.bmj.com/site/about/guidelines.xhtml

Figure 3. Age-related vascular calcification in never smoker with or without secondhand smoke exposure

Secondhand smoking exposure was defined by self-reports (A), serum cotinine (B), and urinary NNAL levels (C), respectively. The spline curve shows the relationship between AAC-24 scores and age in never smokers with or without SHS exposure based on fractional polynomial regression.

For peer review only

For peer review only - http://bmjopen.bmj.com/site/about/guidelines.xhtml

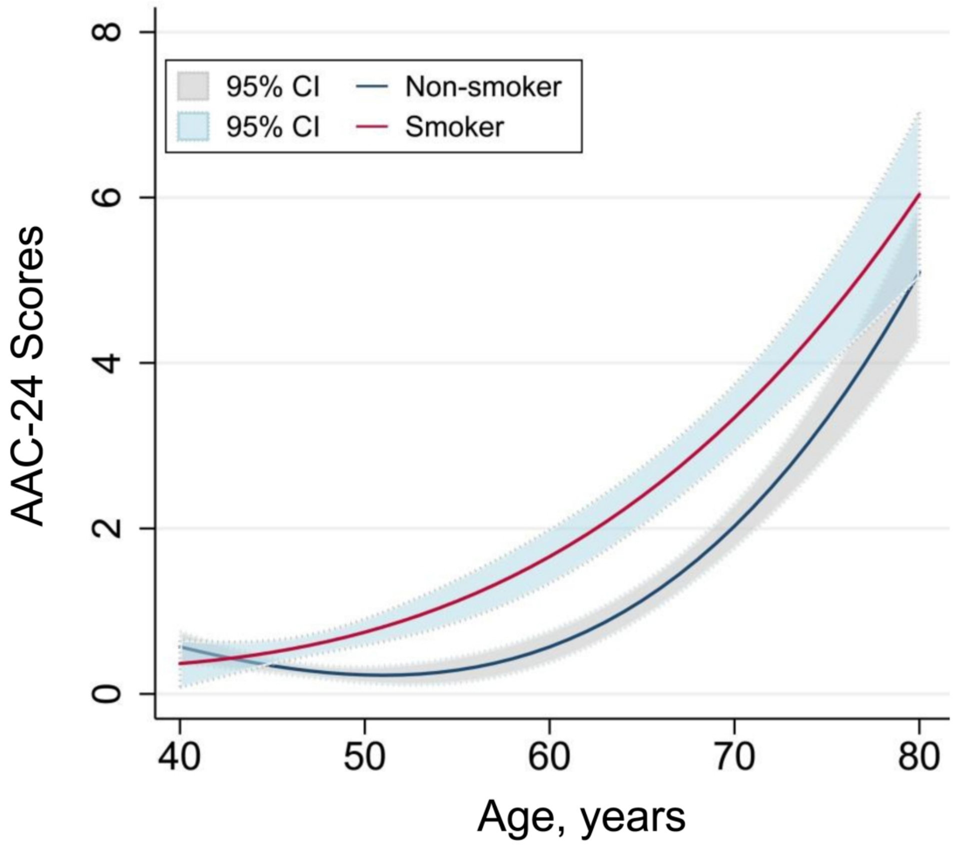

Figure 1. Spline fitting of aged-related AAC in nonsmokers and smokers

112x98mm (1200 x 1200 DPI)

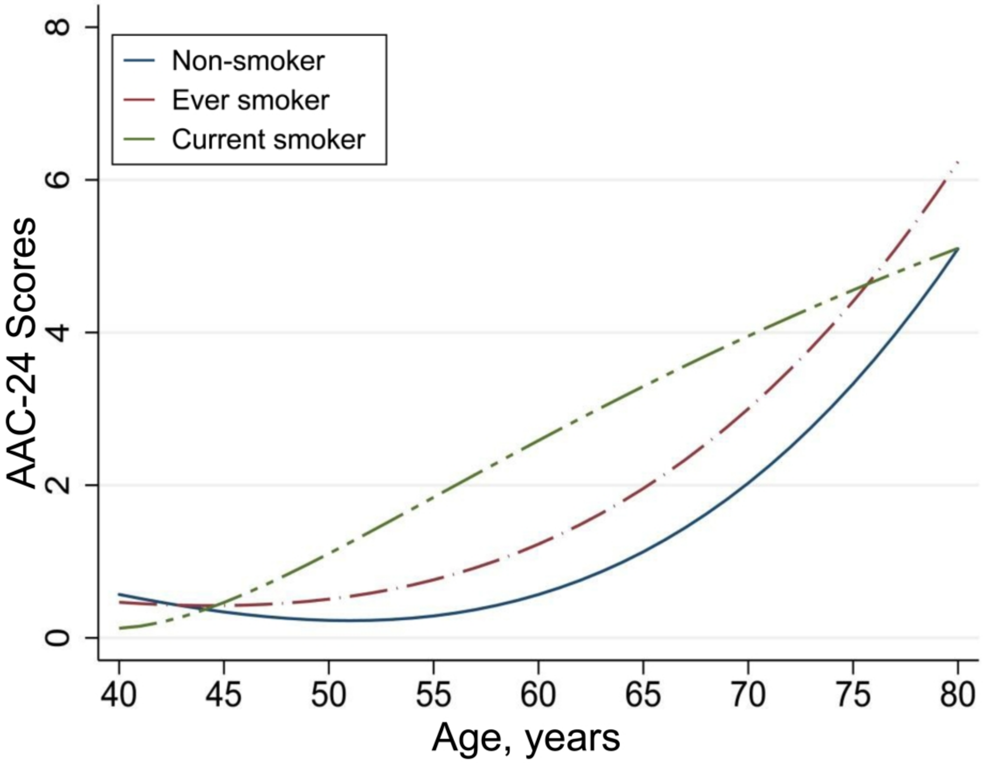

Figure 2. The visualization of age-related AAC in smokers with quit smoking or not

124x96mm (1200 x 1200 DPI)

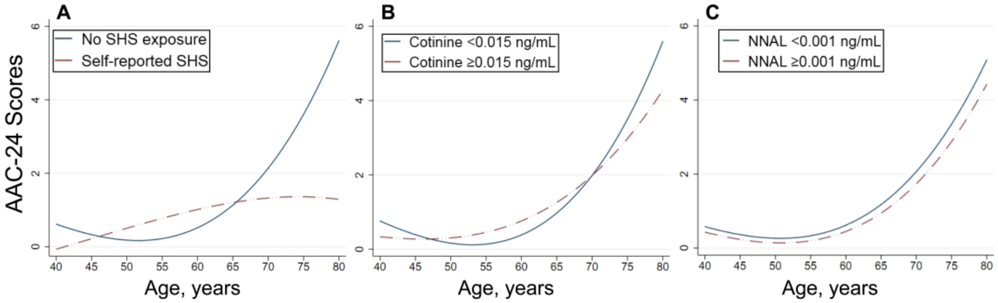

Figure 3. Age-related vascular calcification in nonsmokers with or without secondhand smoking exposure

184x56mm (1200 x 1200 DPI)

Supplemental data

Supplemental Table 1. Association between age and AAC scores in overall or subgroups by smoking

Models	Overall		Never smoker		Smoker		p for interaction
**AAC-24**	β coefficient (95%CI)	p value	β coefficient (95%CI)	p value	β coefficient (95%CI)	p value	
Crude	0.57 (0.49-0.66)	<0.001	0.49 (0.42-0.57)	<0.001	0.66 (0.53-0.80)	<0.001	0.018
Model 1	0.57 (0.49-0.66)	<0.001	0.50 (0.42-0.58)	<0.001	0.66 (0.52-0.79)	<0.001	0.018
Model 2	0.43 (0.33-0.52)	<0.001	0.37 (0.29-0.44)	<0.001	0.53 (0.38-0.68)	<0.001	0.005
**AAC-8**							
Crude	0.20 (0.17-0.23)	<0.001	0.18 (0.15-0.21)	<0.001	0.22 (0.18-0.26)	<0.001	0.081
Model 1	0.20 (0.17-0.23)	<0.001	0.19 (0.16-0.22)	<0.001	0.22 (0.18-0.26)	<0.001	0.081
Model 2	0.16 (0.12-0.19)	<0.001	0.14 (0.11-0.18)	<0.001	0.18 (0.14-0.23)	<0.001	0.017

β coefficient (95%CI) for AAC scores with a 5-year increase of age. p for interaction was estimated by the survey-weighted Wald test was used to assess the potential interaction between age and smoking for AAC. Model 1 (n=3,140): adjusted for sex, and race/ethnicity. Model 2 (n=2,992): additionally adjusted for body mass index, smoking status, alcohol consumption, physical activity, diabetes, hypertension, TC, TG, HDL-C, eGFR, and lowing lipid agents.

For peer review only - http://bmjopen.bmj.com/site/about/guidelines.xhtml

Supplemental Table 2. Association between active smoking status and AAC scores

	Never smoking	Quit smoking	Current smoking	
**AAC-8**		**β (95%CI)**	**β (95%CI)**	**p for trend**
Crude	Reference	0.31 (0.17-0.45)	0.23 (0.04-0.43)	0.002
Model 1	Reference	0.15 (0.01-0.29)	0.40 (0.22-0.58)	<0.001
Model 2	Reference	0.14 (0.00-0.29)	0.29 (0.12-0.46)	0.003
**AAC-24**		**β (95%CI)**	**β (95%CI)**	
Crude	Reference	0.92 (0.55-1.29)	0.51 (0.08-0.94)	0.001
Model 1	Reference	0.46 (0.12-0.79)	0.96 (0.54-1.38)	<0.001
Model 2	Reference	0.46 (0.10-0.81)	0.70 (0.34-1.05)	0.001
**Severe AAC**		**OR (95%CI)**	**OR (95%CI)**	
Crude	Reference	2.02 (1.51-2.71)	1.59 (0.94-2.68)	0.012
Model 1	Reference	1.66 (1.18-2.35)	4.30 (2.40-7.71)	<0.001
Model 2	Reference	1.69 (1.12-2.56)	3.39 (2.04-5.66)	<0.001
**Subclinical AAC**		**OR (95%CI)**	**OR (95%CI)**	
Crude	Reference	1.85 (1.37-2.49)	1.73 (1.12-2.66)	0.003
Model 1	Reference	1.47 (1.05-2.07)	2.78 (1.82-4.25)	<0.001
Model 2	Reference	1.46 (1.00-2.14)	2.06 (1.34-3.16)	0.004

β coefficient (95%CI) for AAC scores acorss the smoking status with never smoking as reference. Model 1 (n=3,140): adjusted for age, sex, and race/ethnicity. Model 2 (n=2,992): additionally adjusted for body mass index, alcohol consumption, physical activity, diabetes, hypertension, TC, TG, HDL-C, eGFR, and lowing lipid agents.

For peer review only - http://bmjopen.bmj.com/site/about/guidelines.xhtml

Supplemental Table 3. Association between smoking cessation and AAC scores in smokers

	**Current smoking** OR (95%CI)	**Smoking Cessation <10y** OR (95%CI)	**Smoking Cessation ≥10y** OR (95%CI)	**p for trend**
**Subclinical AAC**	Reference	0.97 (0.63-1.48)	0.65 (0.44-0.94)	0.023
**Severe AAC**	Reference	0.96 (0.40-2.34)	0.37 (0.20-0.69)	0.003

OR (95%CI) for the risk of severe AAC or subclinical AAC was estimated by weighted logistics regression analysis, adjusted for age, sex, race/ethnicity, body mass index, alcohol consumption, physical activity, diabetes, hypertension, TC, TG, HDL-C, eGFR, and lowing lipid agents.

For peer review only - http://bmjopen.bmj.com/site/about/guidelines.xhtml

Supplemental Table 4. Association between secondhand smoking exposure (SHS) and AAC in never smoker

SHS	Subclinical AAC OR (95%CI)	p value	Severe AAC OR (95%CI)	p value
self-report	0.96 (0.20-4.71)	0.958	0.61 (0.29-1.30)	0.183
NNAL	0.61 (0.30-1.26)	0.167	0.73 (0.51-1.03)	0.073
Cotinine	0.84 (0.49-1.44)	0.500	0.96 (0.68-1.35)	0.792

SHS exposure, was defined as self-report, urinary NNAL≥0.001ng/mL, or serum cotinine ≥0.015ng/mL. OR (95%CI) for the risk of severe AAC or subclinical AAC was estimated by weighted logistics regression analysis, adjusted for age, sex, race/ethnicity, body mass index, alcohol consumption, physical activity, diabetes, hypertension, TC, TG, HDL-C, eGFR, and lowing lipid agents.

For peer review only

For peer review only - http://bmjopen.bmj.com/site/about/guidelines.xhtml

STROBE Statement—Checklist of items that should be included in reports of ***cross-sectional studies***

	Item No	Recommendation	
**Title and abstract**	1	(*a*) Indicate the study's design with a commonly used term in the title or the abstract	P1
		(*b*) Provide in the abstract an informative and balanced summary of what was done and what was found	P2
**Introduction**			
Background/rationale	2	Explain the scientific background and rationale for the investigation being reported	P5
Objectives	3	State specific objectives, including any prespecified hypotheses	P6
**Methods**			
Study design	4	Present key elements of study design early in the paper	P7
Setting	5	Describe the setting, locations, and relevant dates, including periods of recruitment, exposure, follow-up, and data collection	P7-9
Participants	6	(*a*) Give the eligibility criteria, and the sources and methods of selection of participants	P7
Variables	7	Clearly define all outcomes, exposures, predictors, potential confounders, and effect modifiers. Give diagnostic criteria, if applicable	P8-9
Data sources/ measurement	8*	For each variable of interest, give sources of data and details of methods of assessment (measurement). Describe comparability of assessment methods if there is more than one group	P7
Bias	9	Describe any efforts to address potential sources of bias	P10
Study size	10	Explain how the study size was arrived at	P7
Quantitative variables	11	Explain how quantitative variables were handled in the analyses. If applicable, describe which groupings were chosen and why	P9
Statistical methods	12	(*a*) Describe all statistical methods, including those used to control for confounding	P9-10
		(*b*) Describe any methods used to examine subgroups and interactions	P9-10
		(*c*) Explain how missing data were addressed	
		(*d*) If applicable, describe analytical methods taking account of sampling strategy	
		(*e*) Describe any sensitivity analyses	P9-10
**Results**			
Participants	13*	(a) Report numbers of individuals at each stage of study—eg numbers potentially eligible, examined for eligibility, confirmed eligible, included in the study, completing follow-up, and analysed	P11
		(b) Give reasons for non-participation at each stage	
		(c) Consider use of a flow diagram	
Descriptive data	14*	(a) Give characteristics of study participants (eg demographic, clinical, social) and information on exposures and potential confounders	P11
		(b) Indicate number of participants with missing data for each variable of interest	
Outcome data	15*	Report numbers of outcome events or summary measures	P11-12
Main results	16	(*a*) Give unadjusted estimates and, if applicable, confounder-adjusted estimates and their precision (eg, 95% confidence interval). Make clear which confounders were adjusted for and why they were included	

For peer review only

		(*b*) Report category boundaries when continuous variables were categorized	P11-12
		(*c*) If relevant, consider translating estimates of relative risk into absolute risk for a meaningful time period	P11-12
Other analyses	17	Report other analyses done—eg analyses of subgroups and interactions, and sensitivity analyses	
**Discussion**			
Key results	18	Summarise key results with reference to study objectives	P13
Limitations	19	Discuss limitations of the study, taking into account sources of potential bias or imprecision. Discuss both direction and magnitude of any potential bias	P16
Interpretation	20	Give a cautious overall interpretation of results considering objectives, limitations, multiplicity of analyses, results from similar studies, and other relevant evidence	P13-15
Generalisability	21	Discuss the generalisability (external validity) of the study results	P16
**Other information**			
Funding	22	Give the source of funding and the role of the funders for the present study and, if applicable, for the original study on which the present article is based	

*Give information separately for exposed and unexposed groups.

Note: An Explanation and Elaboration article discusses each checklist item and gives methodological background and published examples of transparent reporting. The STROBE checklist is best used in conjunction with this article (freely available on the Web sites of PLoS Medicine at http://www.plosmedicine.org/, Annals of Internal Medicine at http://www.annals.org/, and Epidemiology at http://www.epidem.com/). Information on the STROBE Initiative is available at www.strobe-statement.org.

For peer review only
